# Supplementary material for: Suppressing electron-phonon coupling in organic photovoltaics for high-efficiency power conversion
Source: Nat Commun. 2023 Aug 21;14:5079. doi: 10.1038/s41467-023-40806-9 (PMC10442373; doi:10.1038/s41467-023-40806-9)
Supplement: Supplementary file 1 — Supplementary Information [file 41467_2023_40806_MOESM1_ESM.pdf]

Supplementary information

Suppressing Electron-Phonon Coupling in Organic Photovoltaics for High-Efficiency Power Conversion

Yuanyuan Jiang,[1,2,4] Yixin Li,[1,4] Feng Liu,[1,4] Wenxuan Wang,[1,2] Wenli Su,[3] Wuyue Liu,[1] Songjun Liu,[1,2] Wenkai Zhang,[3] Jianhui Hou,[1,2] Shengjie Xu,[1*] Yuanping Yi[1*] & Xiaozhang Zhu[1,2*]

[1]Beijing National Laboratory for Molecular Sciences, CAS Key Laboratory of Organic Solids, Institute of Chemistry, Chinese Academy of Sciences, Beijing 100190, China

[2]School of Chemical Sciences, University of Chinese Academy of Sciences, Beijing 100049, China

[3]Department of Physics and Applied Optics, Beijing Area Major Laboratory Center for Advanced Quantum Studies, Beijing Normal University, Beijing 100875, China.

[4]These authors contributed equally: Yuanyuan jiang, Yixin Li, Feng Liu

For correspondence: *xushengjie@iccas.ac.cn, *ypyi@iccas.ac.cn, *xzzhu@iccas.ac.cn

Contents

1. **Materials and Synthesis Procedures**

2. **Supplementary Figures**

3. **Supplementary Tables**

1. Materials and Synthesis Procedures

General Methods

All reactions involving air- or moisture-sensitive compounds were carried out in a dry reaction vessel under a positive pressure of nitrogen. **AQx-2** was prepared according to our published procedures (*Adv. Mater.*, **2020**, *32*, 1906324). ^{1}H and ^{13}C spectra were measured with Bruker Avance 400 spectrometers. Chemical shifts for hydrogens are reported in parts per million (ppm, scale) downfield from tetramethylsilane and are referenced to the residual protons in the NMR solvent ($CDCl_3$: 7.26).^{13}C NMR spectra were recorded at 100 MHz. Chemical shifts for carbons are reported in parts per million (ppm, scale) downfield from tetramethylsilane and are referenced to the carbon resonance of the solvent ($CDCl_3$: 77.2). The data are presented as follows: chemical shift, multiplicity (s = singlet, d = doublet, t = triplet, m = multiplet and/or multiple resonances, br = broad), coupling constant in hertz (Hz), and integration. MALDI measurements were performed with a MALDI-FT 9.4 T, Bruker solariX, or MALDI-TOF MS Bruker Autoflex III. UV-vis was recorded with Jasco V-570 spectrometers. Cyclic voltammetry (CV) was performed with a CHI620D potentiostat. All measurements were carried out in a one-compartment cell under a nitrogen atmosphere, equipped with a glassy-carbon electrode, a platinum counter-electrode, and an Ag/Ag^{+} reference electrode with a scan rate of 100 mV/s. The supporting electrolyte was a 0.1 mol/L dichloromethane solution of tetrabutylammonium perchlorate (TBAP). All potentials were corrected against Fc/Fc^{+}. Single crystals were grown through slow diffusion of ethanol to its chloroform solution.

Compound 2. Compound 1 (0.374 g, 0.5 mmol) was dissolved in 20 mL N,N-dimethylformamide (DMF) and added 1-Bromo-2-ethylhexane (0.145 g, 0.75 mmol, 1.5 eq.) and Potassium carbonate (K_2CO_3, 0.345 g, 2.5 mmol, 5 eq.) under a nitrogen atmosphere. The reaction mixture was then heated at 80 °C for two hours, quenched by addition of water and extracted with ethyl acetate. The combined extracts were washed with brine, and then filtered and collected solvent. The solvent was removed by rotary evaporation to give the crude product, which was further purified by column chromatography on silica gel to afford compound 2 as a red brown solid (0.301 g, 70%). ^{1}H NMR (400 MHz, $CDCl_3$): δ 8.09 (s, 1H), 6.83 (m, 2H), 3.80 (m, 2H), 2.60 (m, 4H), 1.20−0.50 (m, 57H); ^{13}C NMR (100 MHz, $CDCl_3$): δ 147.0, 146.6, 142.0, 141.6, 136.6, 136.4, 134.9, 131.6, 127.0, 126.8, 122.4, 122.1, 121.9, 119.8, 119.1, 118.9, 109.4, 108.9, 52.4, 41.6, 39.4, 39.1, 38.9, 38.8, 37.0, 36.6, 36.5, 36.4, 34.5, 34.3, 33.1, 32.4, 32.0, 31.1, 30.3, 30.0, 29.9, 29.7, 29.6, 29.5, 29.4, 29.0, 28.7, 28.4, 28.0, 27.9, 27.5, 27.4, 27.3, 27.2, 27.1, 27.0, 26.6, 26.3, 25.3, 24.7, 23.1, 23.0, 22.7, 22.3, 20.4, 20.2, 19.7, 19.3, 19.2, 18.1, 14.5, 14.2, 14.1, 11.5, 11.4, 11.2, 11.0; MS (MALDI-TOF) $C_{48}H_{66}N_4S_5$ [M]+: 858.3.

Compound 3. Compound 2 (0.25 g, 0.29 mmol) was dissolved in 15 mL DMF and added 2-hexyldecylbromide (0.442 g, 1.45 mmol, 5 eq.) and potassium carbonate (K_2CO_3, 0.4 g, 2.9 mmol, 10 eq.) under a nitrogen atmosphere. The reaction mixture was then heated at 140 °C for four hours, quenched by addition of water and extracted with ethyl acetate. The combined extracts were washed with brine, and then filtered and collected solvent. The solvent was removed by rotary evaporation to give the crude

product, which was then dissolved in anhydrous acetic acid (15 mL) and added zinc powder (0.188 g, 2.9 mmol, 10 eq.) under a nitrogen atmosphere. The reaction mixture was then heated to reflux for one hour, After the solution was cooled to room temperature, the solid was removed by filtration. The mixture solution was added with water, extracted with ethyl acetate and sodium hydroxide solution until the organic layer was basic. The combined organic layer was dried over $MgSO_4$. After removal of the solvent under reduced pressure, a blown liquid was obtained without further purification. To solution of blown liquid and glyoxal solution (0.29 mmol, 40 wt.% in water) in 20 mL ethanol, the mixture solution was stirred at room temperature for two hours. The solvent was evaporated, and the residue was purified on a silica-gel column to give compound 3 (0.087 g, 28%, three steps). ^{1}H NMR (400 MHz, $CDCl_3$): δ 8.93 (s, 2H), 7.02 (s, 1H), 7.01 (s, 1H), 4.68 (m, 4H), 2.85 (m, 4H), 2.11 (m, 2H), 1.88 (m, 4H), 1.50-0.50 (m, 82H); ^{13}C NMR (100 MHz, CDCl3): δ 143.0, 141.4, 137.6, 137.5, 136.9, 136.4, 130.9, 130.8, 123.7, 123.6, 122.0, 121.9, 119.1, 117.7, 55.0, 55.0, 40.0, 39.0, 38.8, 38.6, 36.5, 34.3, 31.9, 31.8, 31.6, 30.4, 30.0, 29.9, 29.7, 29.6, 29.5, 29.4, 29.3, 29.2, 29.1, 29.0, 28.0, 27.9, 27.7, 27.3, 27.2, 26.8, 26.5, 25.5, 25.4, 23.1, 22.8, 22.7, 22.6, 22.5, 22.4, 19.2, 15.4, 14.4, 14.1, 14.0, 13.7, 11.4, 10.1; MS (MALDI-TOF) $C_{66}H_{100}N_4S_4$ [M]+: 1076.5.

Compound 4. Compound 3 (0.080 g, 0.074 mmol) was dissolved in 10 mL anhydrous THF under nitrogen atmosphere and cooled to –78 °C, then LDA (1 M in THF, 0.3 mL, 0.3 mmol) was added by using a syringe under stirring. After stirring at –78 °C for 2 hours, DMF (0.1 mL) was added and the mixture was stirred at –78 °C for 30 min. The

reaction solution was warmed to room temperature for 30 min, then poured into water and extracted three times with CH_2Cl_2. The organic layer was separated, dried over $MgSO_4$, and concentrated under reduced pressure. The residue was purified by silica-gel column chromatography to give compound 4 (0.078 g, 93%). ^{1}H NMR (400 MHz, $CDCl_3$): δ 10.15 (s, 2H), 8.96 (s, 2H), 4.69 (m, 4H), 3,20 (m, 4H), 2.08 (m, 2H), 1.94 (m, 4H), 1.50-0.50 (m, 82H); ^{13}C NMR (100 MHz, $CDCl_3$): δ 181.8, 146.9, 144.1, 142.2, 137.3, 137.2, 136.9, 136.6, 132.0, 131.9, 129.7, 129.6, 126.8, 126.8, 119.4, 118.4, 118.4, 65.8, 55.3, 55.2, 40.6, 40.2, 38.9, 38.6, 36.6, 34.3, 31.9, 31.9, 31.7, 31.5, 31.0, 30.7, 30.4, 30.1, 29.8, 29.7, 29.7, 29.6, 29.5, 29.4, 29.3, 29.2, 29.1, 28.5, 28.3, 28.1, 27.9, 27.6, 27.5, 26.9, 26.8, 25.5, 23.1, 22.7, 22.6, 22.5, 22.4, 19.1, 14.1, 13.9, 13.7, 11.4, 10.1; MS (MALDI-TOF) $C_{68}H_{100}N_4O_2S_4$ [M]+: 1133.5.

Compound AQx-6. INCN-2F (76 mg, 0.33 mmol) and compound 4 (75 mg, 0.066 mmol) were added to a solvent mixture of chloroform (10 mL) and pyridine (5 drops). The reaction was placed in an oil bath at 65 °C and was stirred for overnight. Removed solvent by reduced pressure and the residue was purified on a silica-gel column chromatography using chloroform as eluent to give AQx-6 as black solid (95 mg, 92%). ^{1}H NMR (400 MHz, $CDCl_3$): δ 9.13 (s, 1H), 9.11 (s, 1H), 8.92 (m, 2H), 8.50 (m, 2H), 7.70 (m, 2H), 4.84 (m, 4H), 3.22 (m, 4H), 2.20 (m, 2H), 1.87 (m, 4H), 1.50-0.50 (m,82H); ^{13}C NMR (150 MHz, $CDCl_3$): δ 186.2, 155.7, 155.6, 154.1, 153.2, 153.0, 146.2, 142.8, 138.2, 138.2, 136.9, 136.7, 136.2, 136.1, 135.3, 135.2, 134.6, 133.2, 133.1, 133.0, 130.6, 130.4, 119.8, 119.7, 119.6, 115.0, 114.8, 114.6, 112.5, 112.3, 68.5, 55.7, 40.5, 39.2, 38.9, 38.7, 36.8, 36.4, 34.4, 34.3, 31.9, 31.8, 31.6, 31.5, 31.4, 30.6, 30.2,

29.9, 29.8, 29.7, 29.6, 29.5, 29.4, 29.3, 29.2, 29.1, 28.0, 27.9, 27.8, 27.7, 27.3, 25.7, 25.6, 23.3, 22.9, 22.7, 22.7, 22.6, 22.5, 20.2, 19.6, 19.2, 14.4, 14.1, 14.0, 13.8, 11.4, 10.3; HRMS (MALDI-TOF) calcd for $C_{92}H_{104}F_4N_8O_2S_4$ [M]+: 1556.7107, found, 1556.7113.

Compound AQx-8. The synthesis process of AQx-8 refers to the synthesis method of AQx-2 that we have reported (*Adv. Mater.*, **2020**, *32*, 1906324). ^{1}H NMR (400 MHz, $CDCl_3$): δ 9.19 (s, 2H), 8.98 (s, 2H), 8.58 (m, 2H), 7.70 (t, ^{3}J = 7.2 Hz, 2H), 4.80 (m, 4H), 3.25 (m, 4H), 2.15 (m, 2H), 1.88 (m, 4H), 1.50-0.50 (m, 98H); ^{13}C NMR (101 MHz, $CDCl_3$): δ 186.1, 158.9, 155.7, 155.7 154.1, 153.1, 153.1, 153.0, 152.9, 146.2, 142.8, 138.1, 136.9, 136.6, 136.6, 136.2, 135.3, 134.5, 133.2, 133.2, 130.7, 119.7, 119.5, 115.1, 115.0, 114.8, 114.6, 112.5, 112.3, 68.4, 55.6, 39.1, 31.9, 31.8, 31.6, 31.5, 31.4, 30.5, 29.9, 29.9, 29.8, 29.7, 29.7, 29.6, 29.5, 29.5, 29.5, 29.4, 29.4, 29.2, 25.6, 25.5, 22.7, 22.6, 22.5, 14.1, 14.1, 14.0; HRMS (MALDI-TOF) calcd for $C_{100}H_{120}F_4N_8O_2S_4$ [M]+: 1668.8348, found, 1668.8335.

2. Supplementary Figures

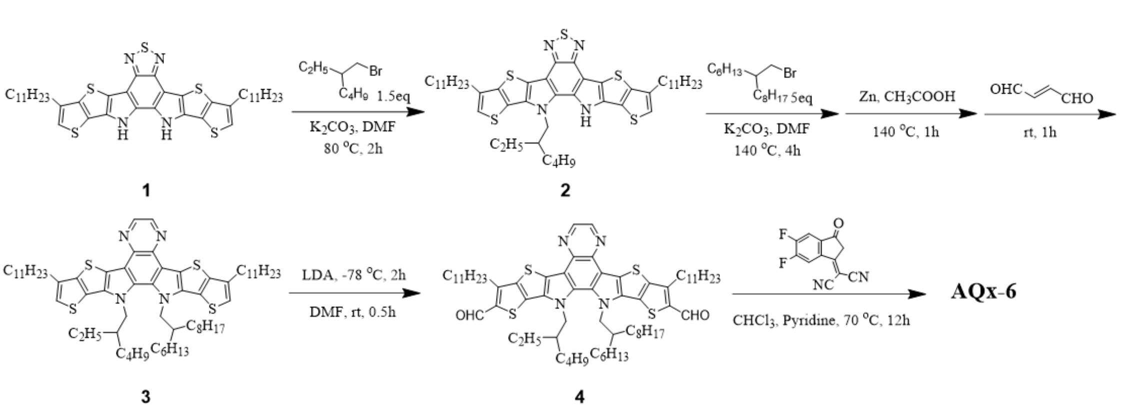

Supplementary Figure 1. The synthesis route of compound AQx-6.

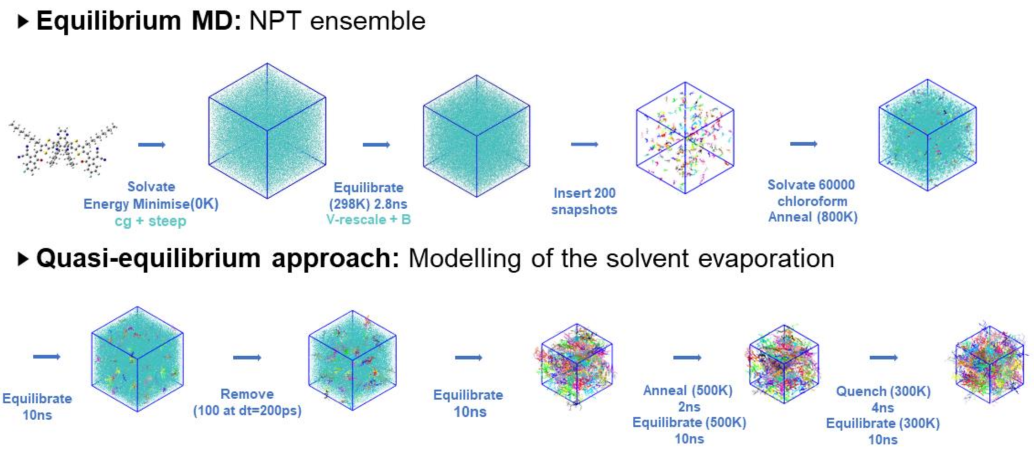

Supplementary Figure 2. The Molecular Dynamics Simulation approaches protocols with the simulated conditions specified and output visualized figure (produced by VMD) in each step.

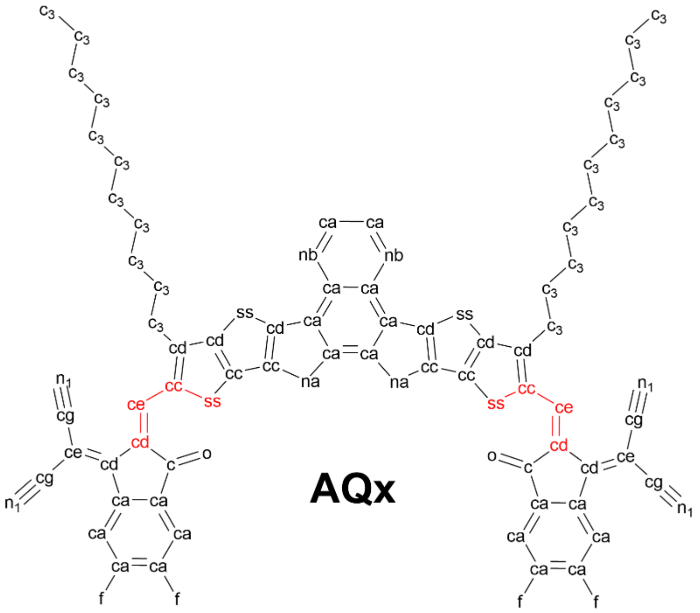

Supplementary Figure 3. Definition of atomic types for AQx from the general AMBER force field (GAFF). The missing torsion potential parameters are highlighted in red. The hydrogen atoms on cd and c3 atoms were defined as ha and hc respectively. The ha and hc hydrogen atoms and regulated alkyl chains were simplified in this figure.

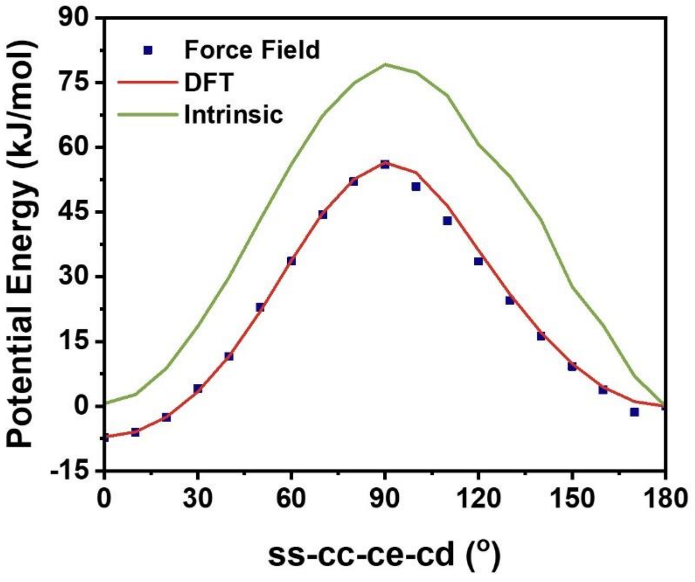

Supplementary Figure 4. AQx-2,-6,-8, were geometry optimized by Gaussian 16 codes under B3LYP-D3(BJ) functional, their two dihedral angles: ss-cc-ce-cd were both fitted using Ryckaert-Bellemans Function, $V_{rb}(\phi_{ijkl}) = \sum_{n=0}^{5} C_n(\cos(\psi))^n$ with the same parameters.

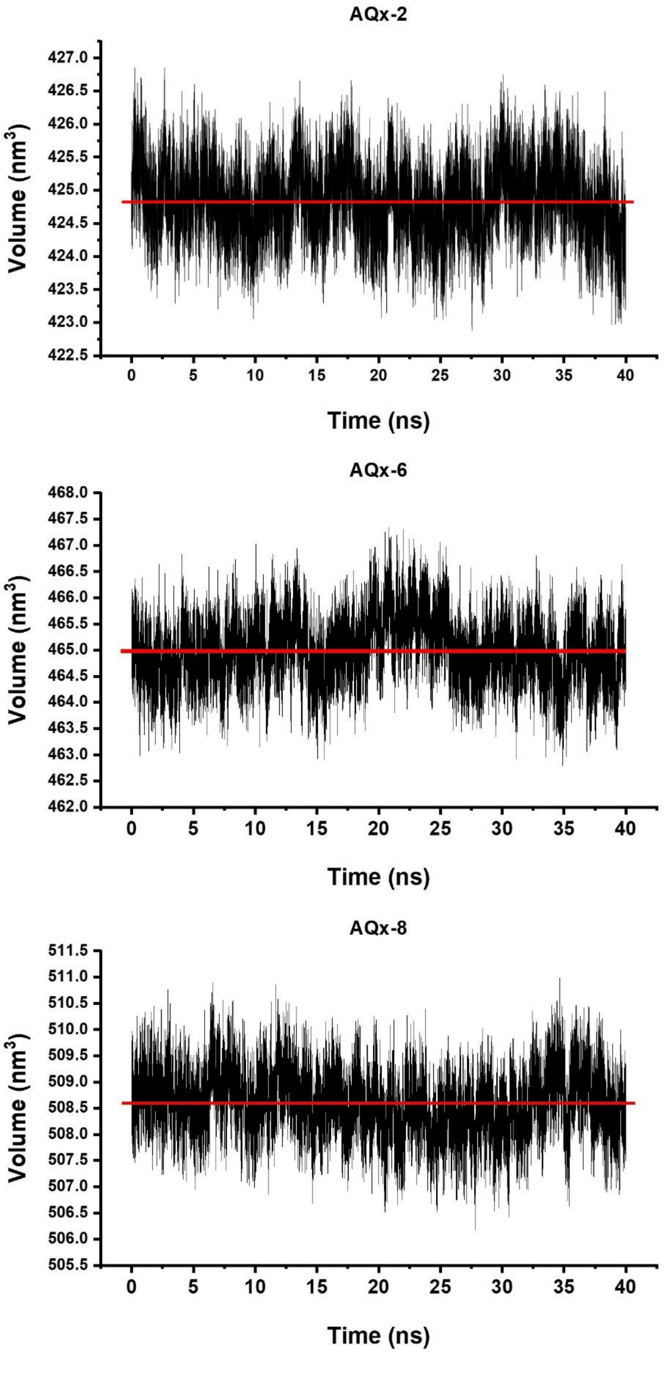

Supplementary Figure 5. The equilibrated 40ns of AQx-2, AQx-6 and AQx-8 after they became thin film (followed the protocol in **Figure S1**). Their volume fluctuations during the equilibrium and their average volume were presented: 424.83 +/- 0.52, 464.99 +/- 0.61, 508.59 +/- 0.60 nm^2

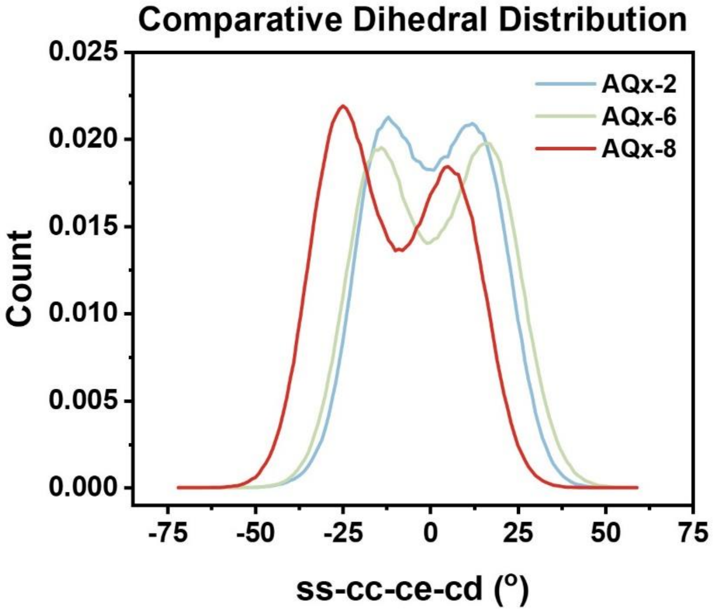

Supplementary Figure 6. Comparative Dihedral Distribution of two ss-cc-ce-cd for all 200 molecules in AQx-2, AQx-6, and AQx-8 thin films during the 40ns equilibrium, data was collected using GROMACS.

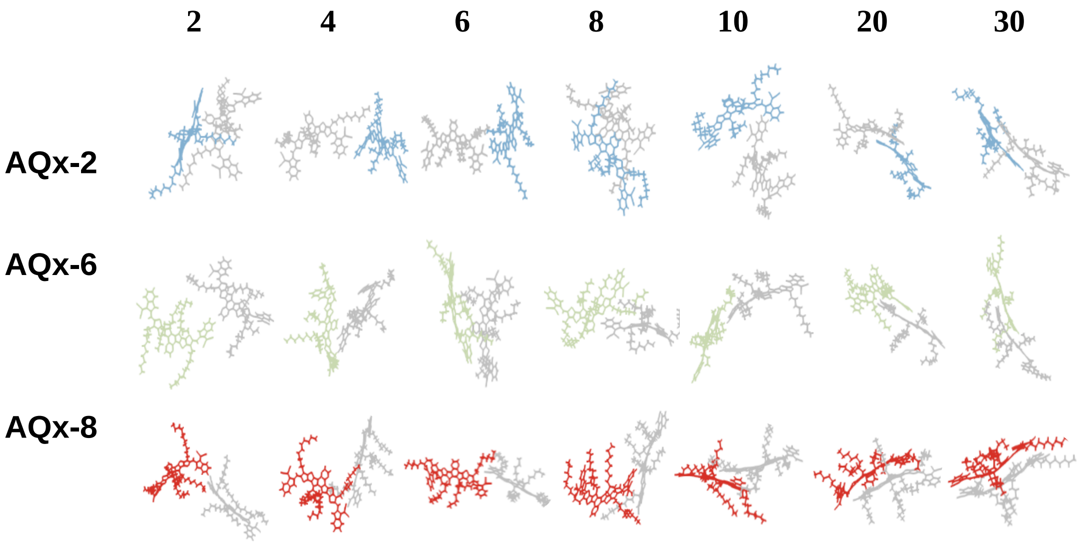

Supplementary Figure 7. The dimer conformations for molecules in AQx-2, AQx-6 and AQx-8 thin film were visualized using VMD as ascending amount of atoms on the molecular backbones contacted.

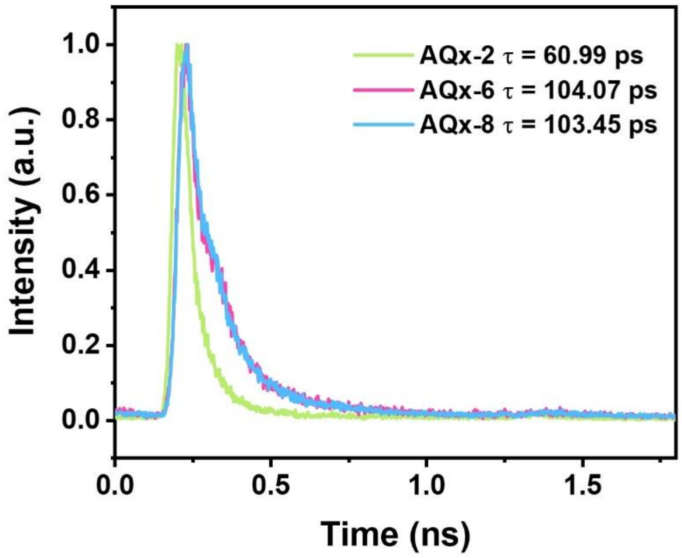

Supplementary Figure 8. The time-resolution photoluminescence (TRPL) spectrum of blend films under the probe of 900 nm (excited at 800 nm).

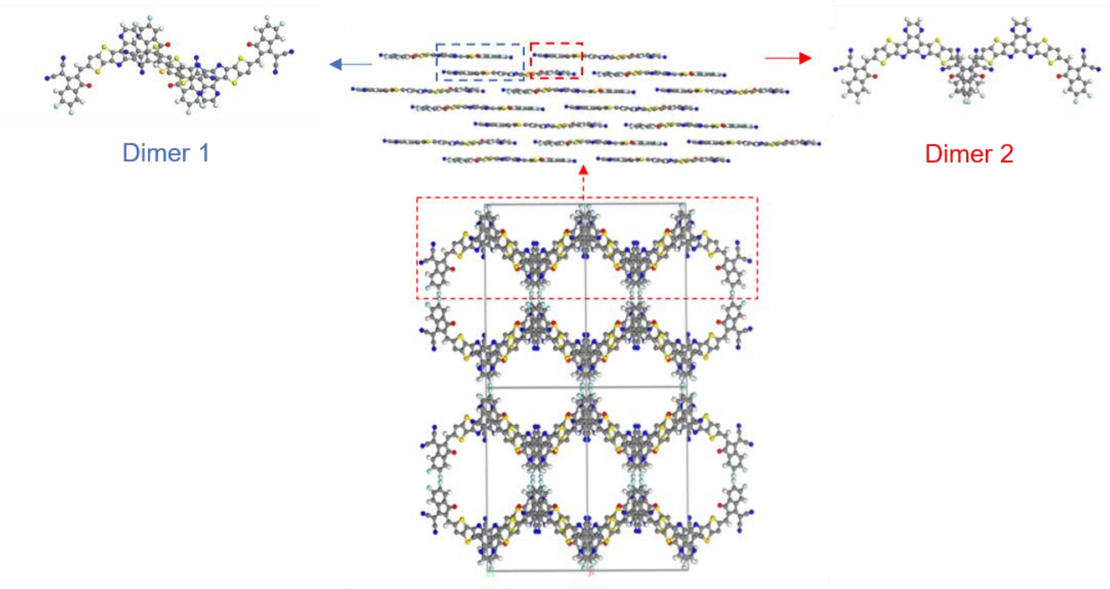

Supplementary Figure 9. The arrangement of the experimental AQx-2 single crystal.

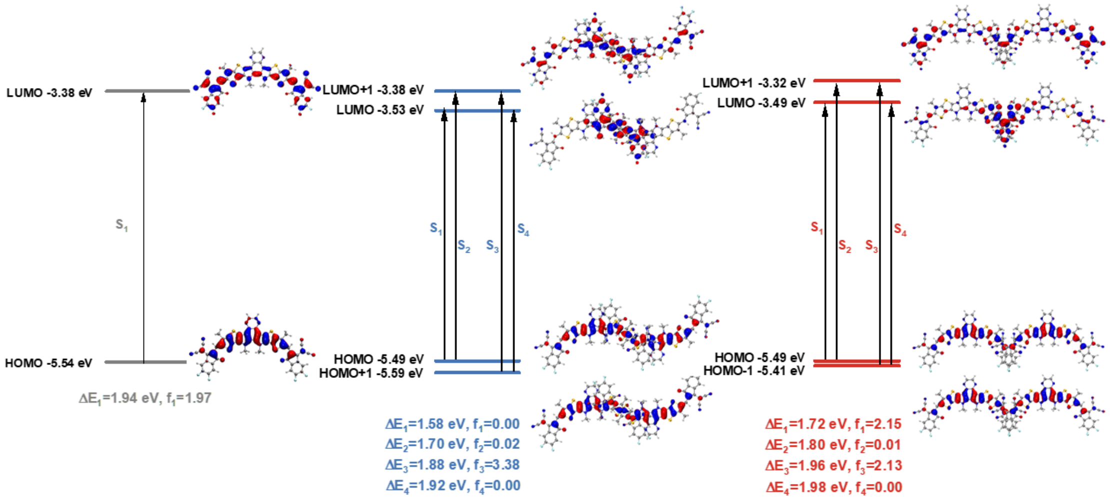

Supplementary Figure 10. The HOMO-LUMO orbital, transition dipole moments, and transition dipole moments of monomer (left), dimer 1 (middle), dimer 2 (right).

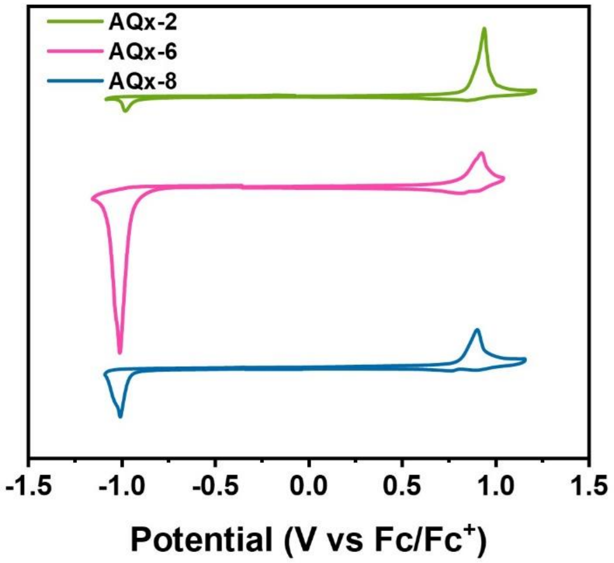

Supplementary Figure 11. Cyclic voltammogram of AQx-2, AQx-6 and AQx-8 film in diluted CH_3CN solution with a scan rate of 100 mV s^{-1}.

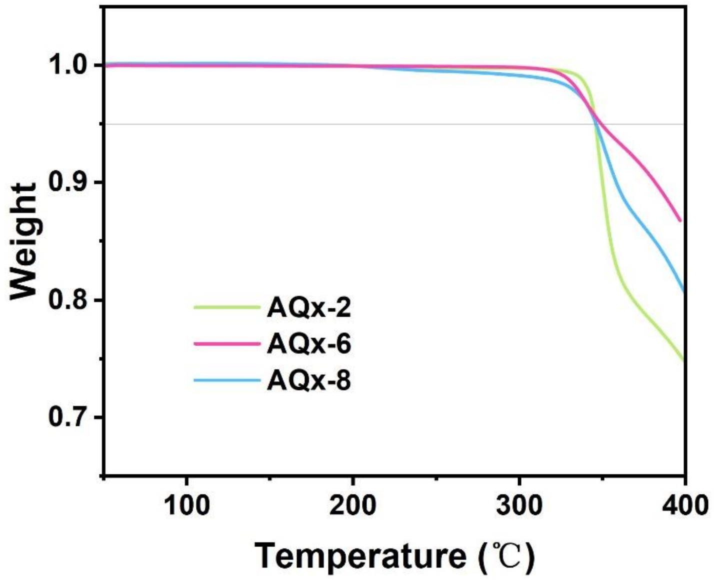

Supplementary Figure 12. The thermogravimetric analysis (TGA) AQx-2, AQx-6 and AQx-8.

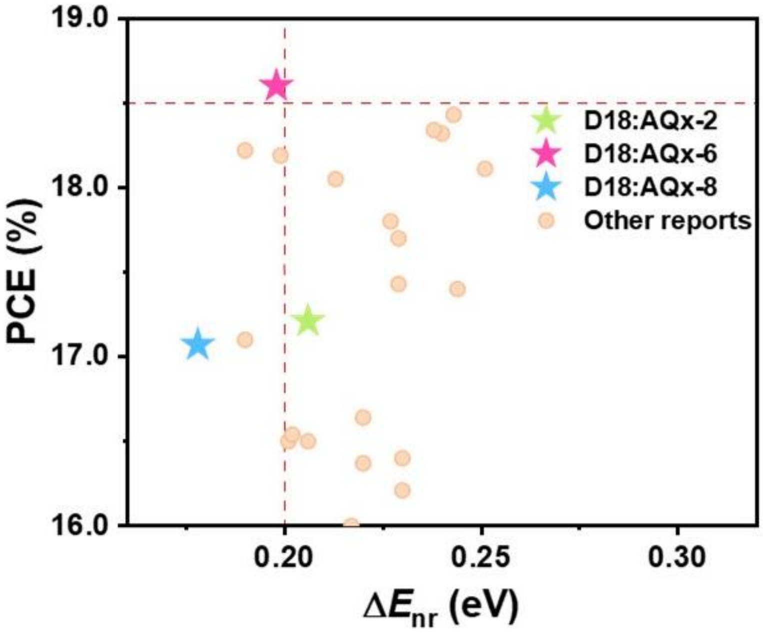

Supplementary Figure 13. Statistics on recent reported bulk-heterojunction binary OSCs.

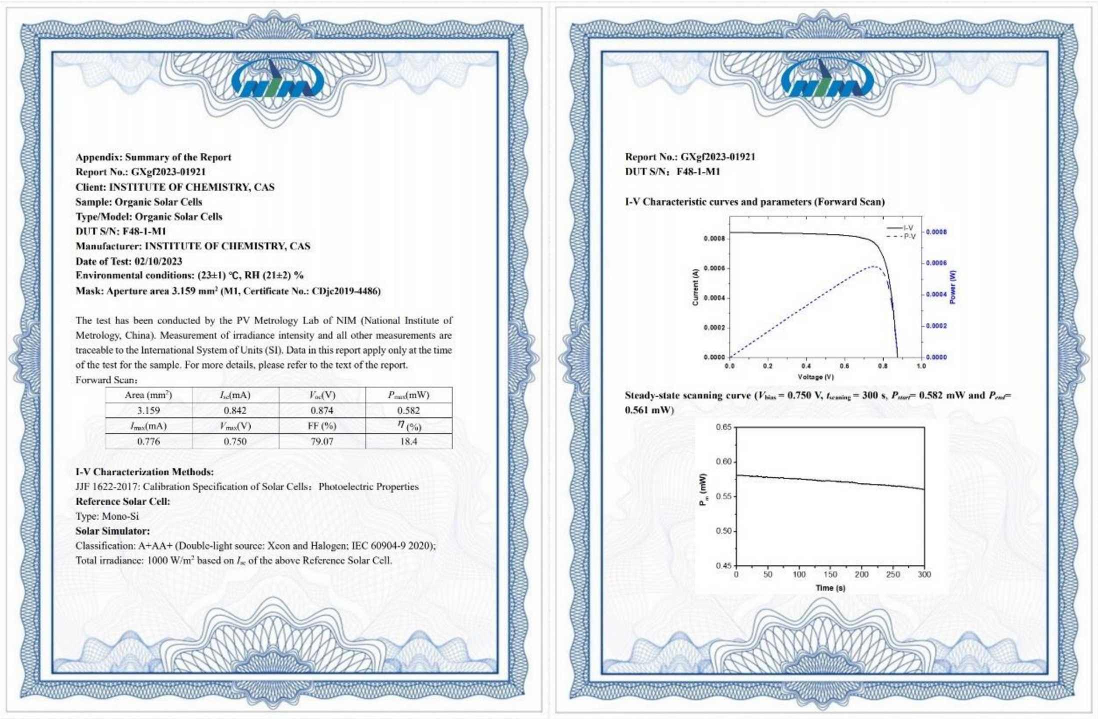

Appendix: Summary of the Report
Report No.: GXgf2023-01921
Client: INSTITUTE OF CHEMISTRY, CAS
Sample: Organic Solar Cells
Type/Model: Organic Solar Cells
DUT S/N: F48-1-M1
Manufacturer: INSTITUTE OF CHEMISTRY, CAS
Date of Test: 02/10/2023
Environmental conditions: (23±1) ℃, RH (21±2) %
Mask: Aperture area 3.159 mm^2 (M1, Certificate No.: CDjc2019-4486)

The test has been conducted by the PV Metrology Lab of NIM (National Institute of Metrology, China). Measurement of irradiance intensity and all other measurements are traceable to the International System of Units (SI). Data in this report apply only at the time of the test for the sample. For more details, please refer to the text of the report.
Forward Scan：

Area (mm^2)	I_{sc}(mA)	V_{oc}(V)	P_{max}(mW)
3.159	0.842	0.874	0.582
I_{max}(mA)	V_{max}(V)	FF (%)	η (%)
0.776	0.750	79.07	18.4

I-V Characterization Methods:
JJF 1622-2017: Calibration Specification of Solar Cells：Photoelectric Properties
Reference Solar Cell:
Type: Mono-Si
Solar Simulator:
Classification: A+AA+ (Double-light source: Xeon and Halogen; IEC 60904-9 2020);
Total irradiance: 1000 W/m^2 based on I_{sc} of the above Reference Solar Cell.

Report No.: GXgf2023-01921
DUT S/N：F48-1-M1

I-V Characteristic curves and parameters (Forward Scan)

Steady-state scanning curve (V_{bias} = 0.750 V, $t_{scaning}$ = 300 s, P_{start}= 0.582 mW and P_{end}= 0.561 mW)

Supplementary Figure 14. The certified PCE for D18:AQx-6, obtained from National Institute of Metrology (NIM), China.

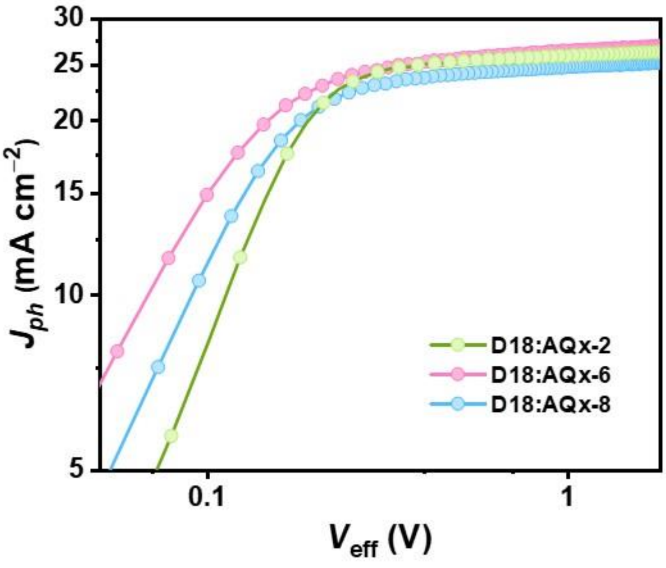

Supplementary Figure 15. J_{ph} versus V_{eff} curves for D18:AQx-2, D18:AQx-6 and D18:AQx-8 devices.

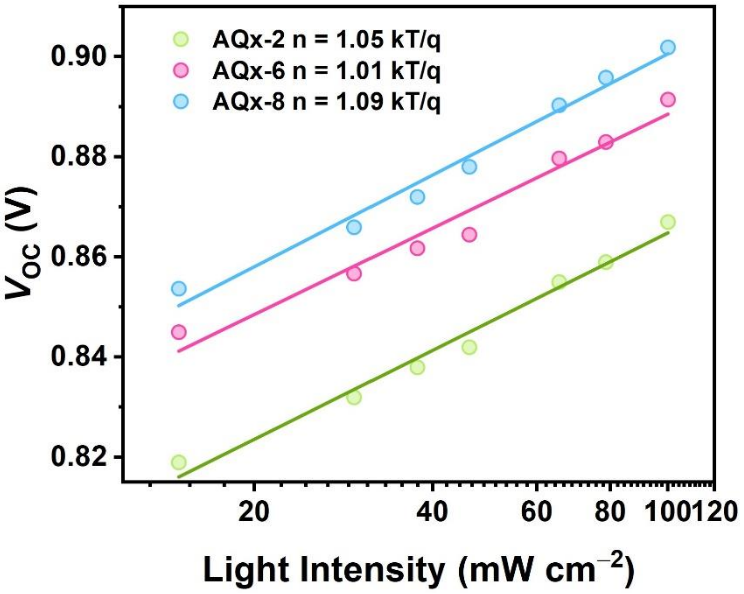

Supplementary Figure 16. The V_{OC} versus light intensity in AQx-2-, AQx-6- and AQx-8-based devices.

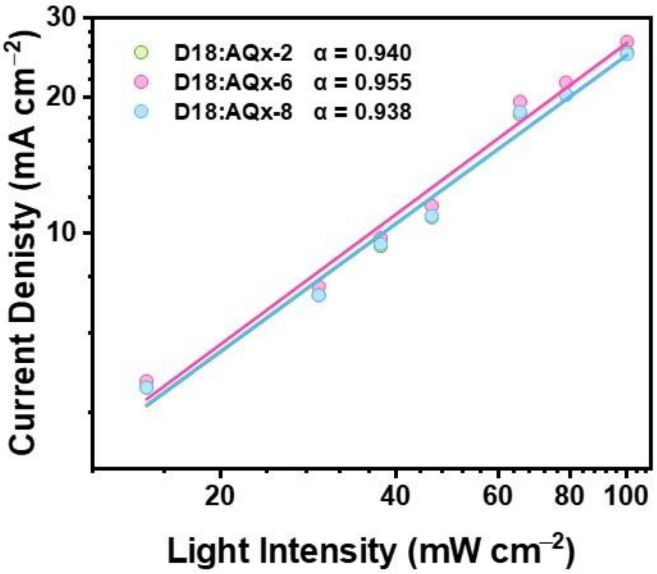

Supplementary Figure 17. J_{sc} dependence on light intensity for D18:AQx-2, D18:AQx-6 and D18:AQx-8 devices.

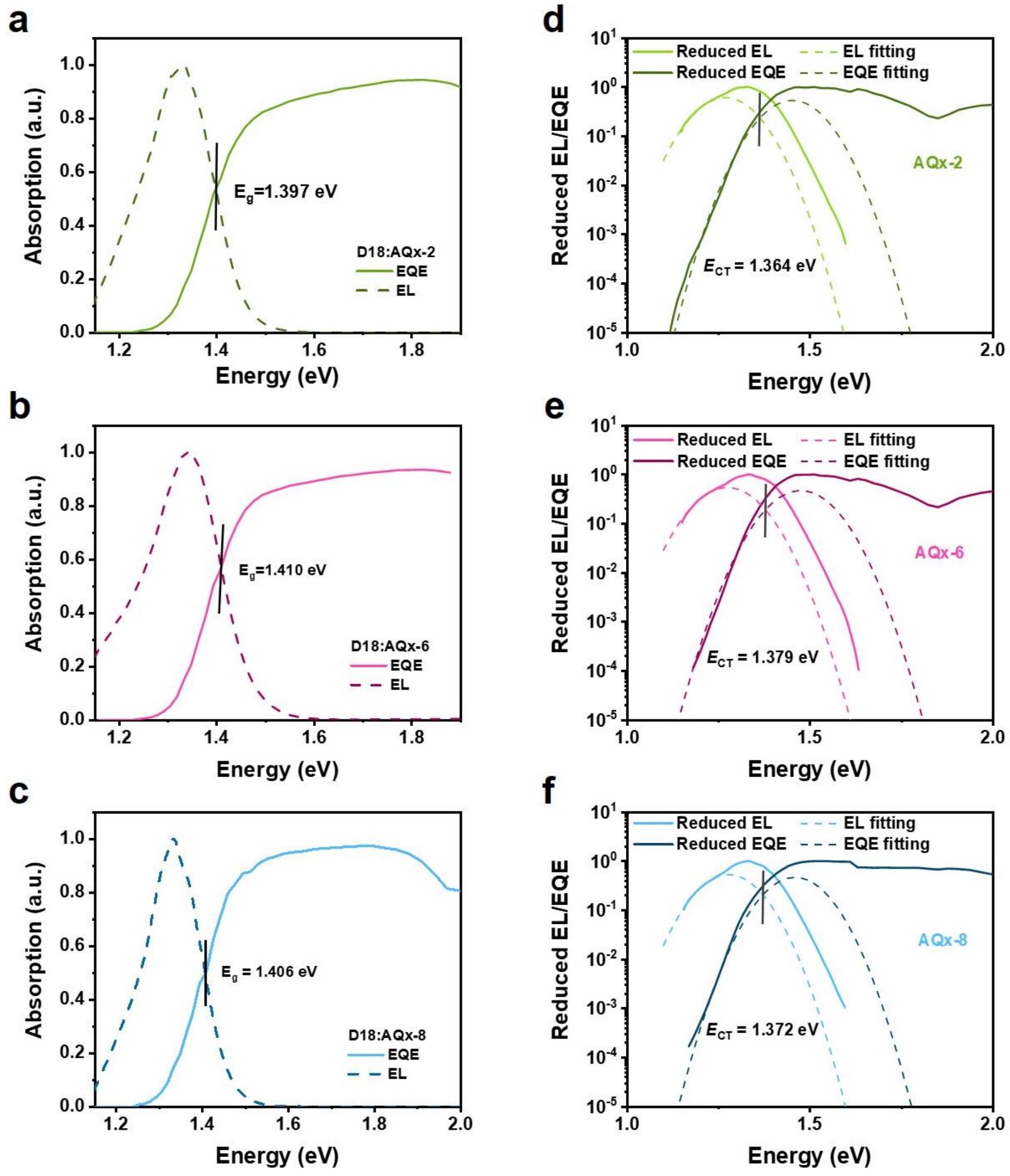

Supplementary Figure 18. a-c) The determination of the E_g of D18:AQx-2, D18:AQx-6 and D18:AQx-8 devices according to the crossing point between the normalized emission and external quantum efficiency spectra, d-f) The determination of the E_{CT} of D18:AQx-2, D18:AQx-6 and D18:AQx-8 devices via fitting reduced EL and sEQE.

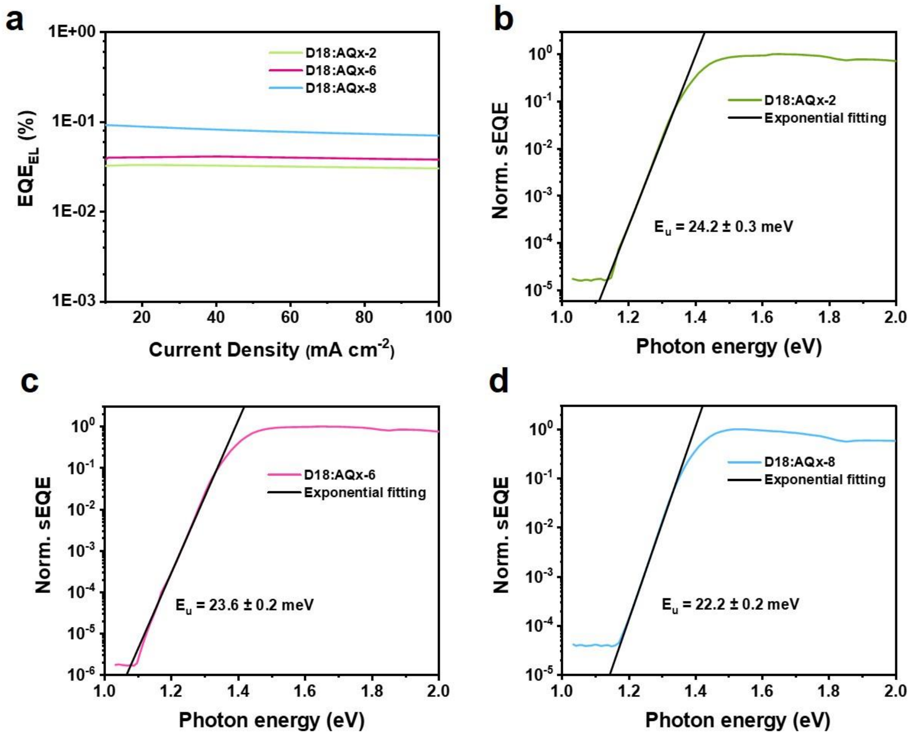

Supplementary Figure 19. a) The EQE$_{EL}$ for D18:AQx-2, D18:AQx-6 and D18:AQx-8 devices, b-d) the highly sensitive EQE spectra of D18:AQx-2, D18:AQx-6 and D18:AQx-8 devices. The Urbach energy (E_u) was fitted by the equation of $E_u = \alpha_0 e^{\frac{E-E_g}{E_U}}$, where α_0 is the optical absorption coefficient at the band edge, E is the photon energy.

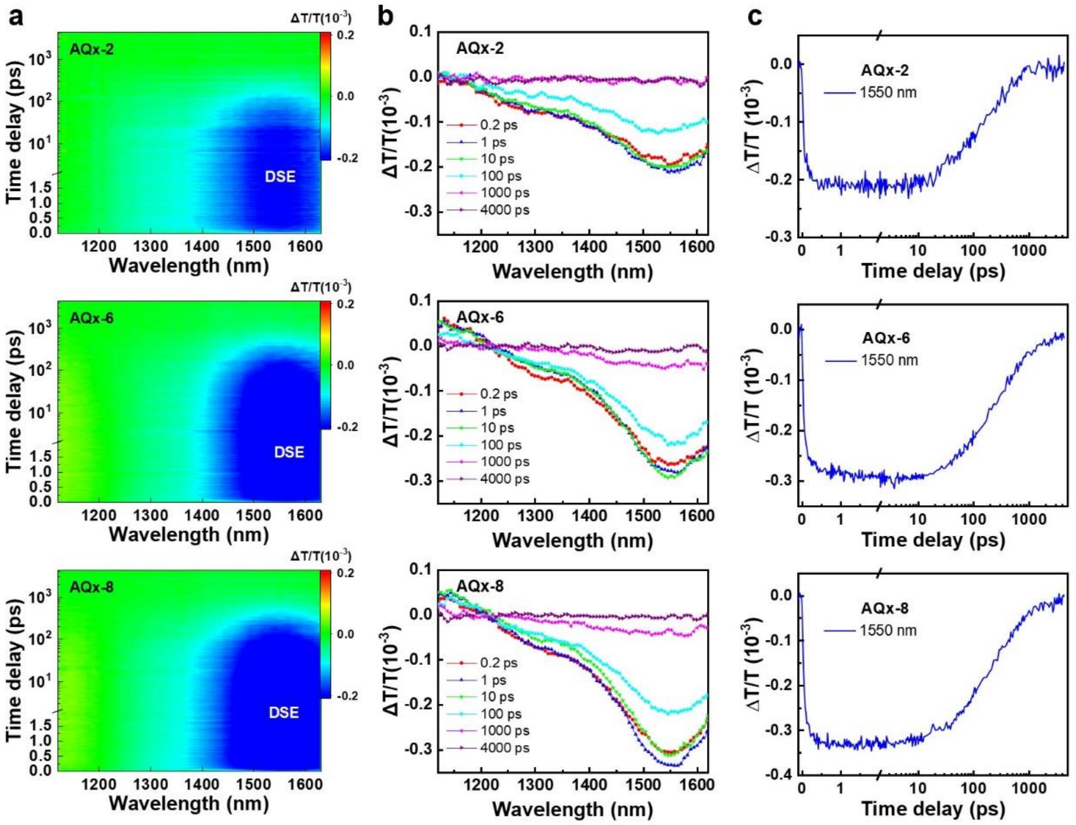

Supplementary Figure 20. a-c, TA profiles (a), TA spectra at different time delay (b) and TA traces of delocalized singlet exciton (DSE) signals probed at 1550 nm (c) for AQx-2, AQx-6 and:AQx-8 neat films with pump at 800 nm.

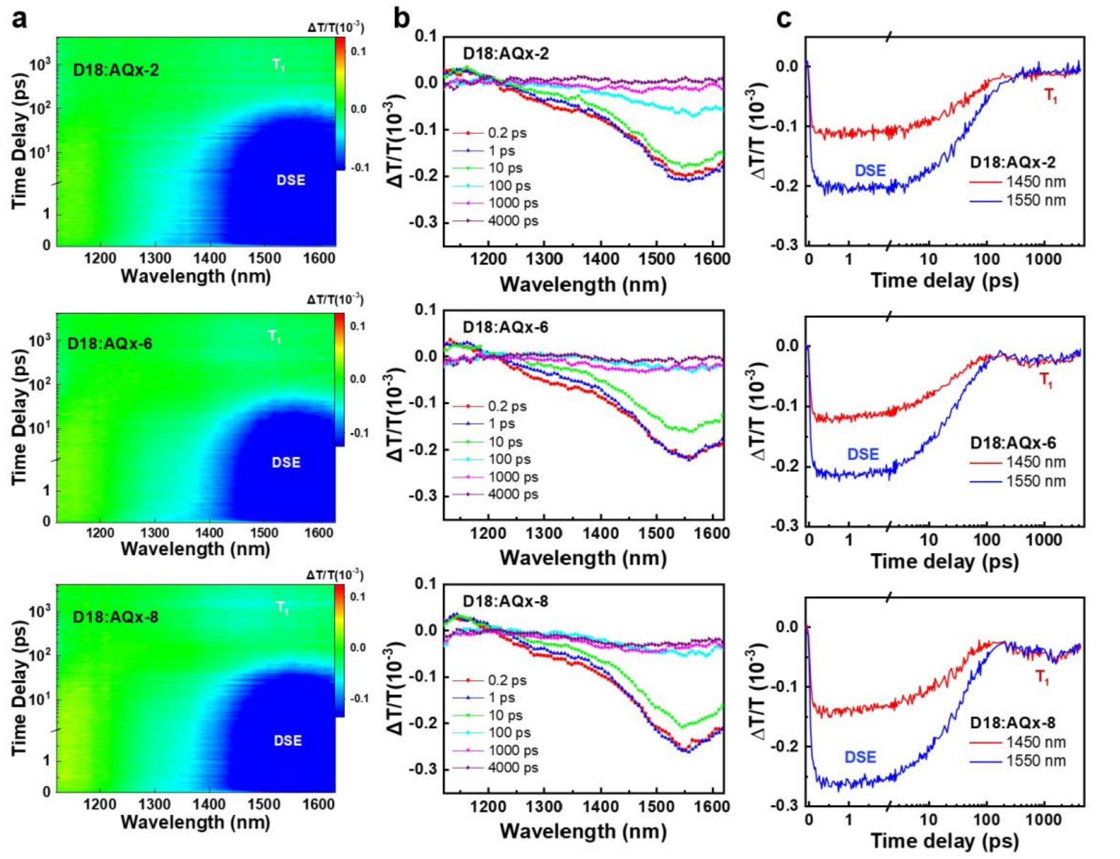

Supplementary Figure 21. a-c, TA profiles (a), TA spectra at different time delay (b) and TA traces of T_1 signals probed at 1450 nm and DSE signals probed at 1550 nm (c) for D18:AQx-2, D18:AQx-6 and D18:AQx-8 blend films with pump at 800 nm.

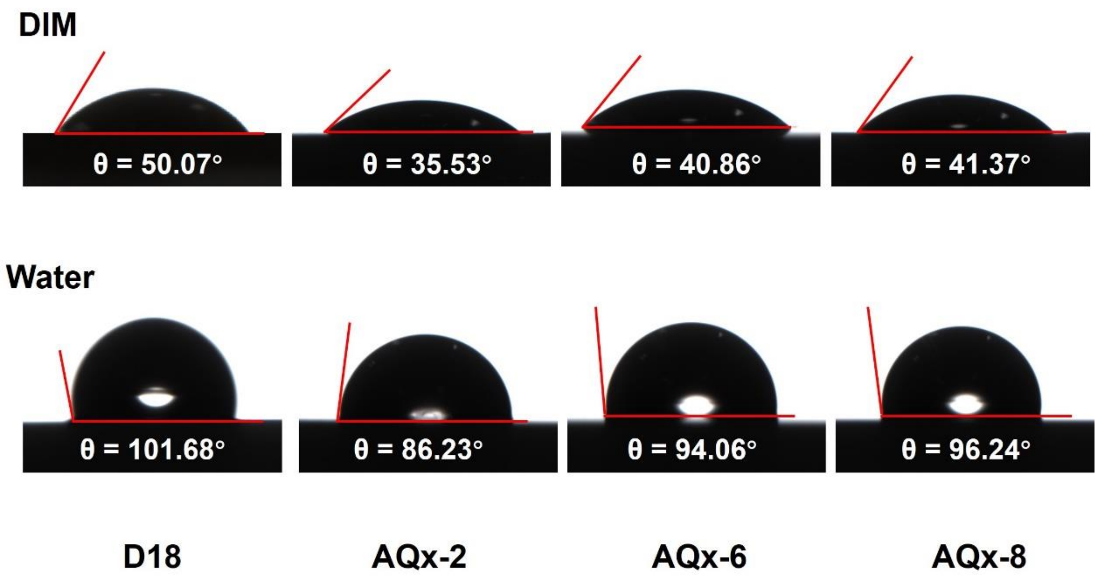

Supplementary Figure 22. The contact angle for D18, AQx-2, AQx-6 and AQx-8. DIM is diiodomethane and water is deionized.

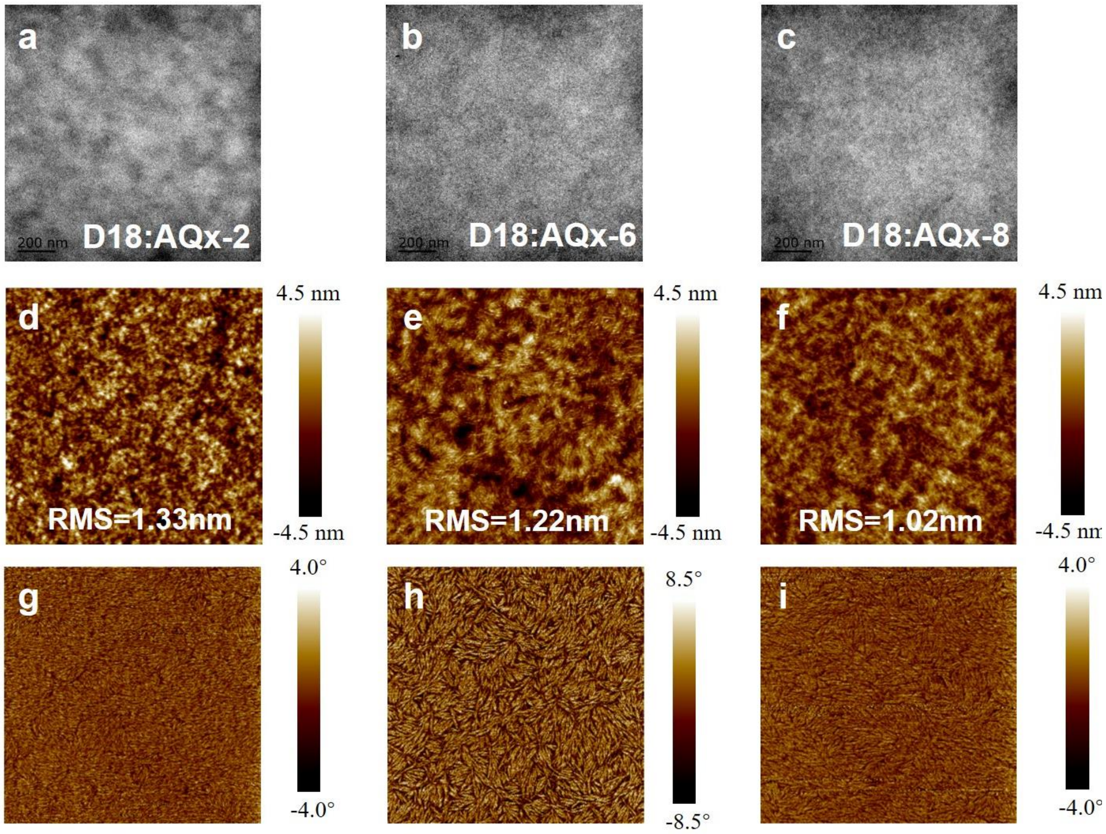

Supplementary Figure 23. a-c) TEM images (200 nm), d-f) AFM height and g-i) phase images of D18:AQx-2, D18:AQx-6 and D18:AQx-8 films (3μm × 3μm).

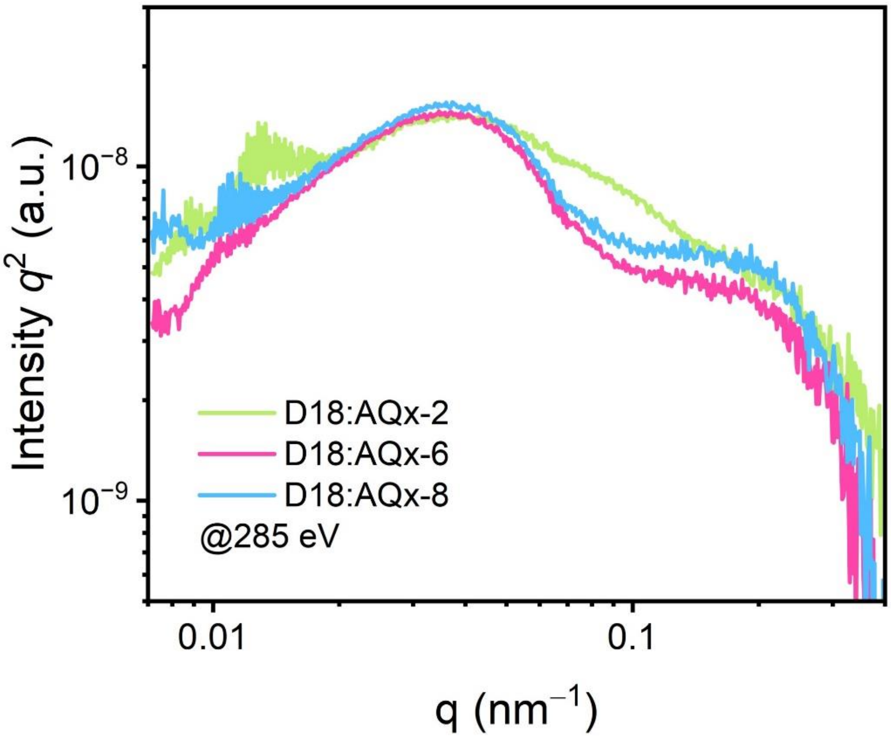

Supplementary Figure 24. RSoXS profiles of D18:AQx-2, D18:AQx-6 and D18:AQx-8 blend films.

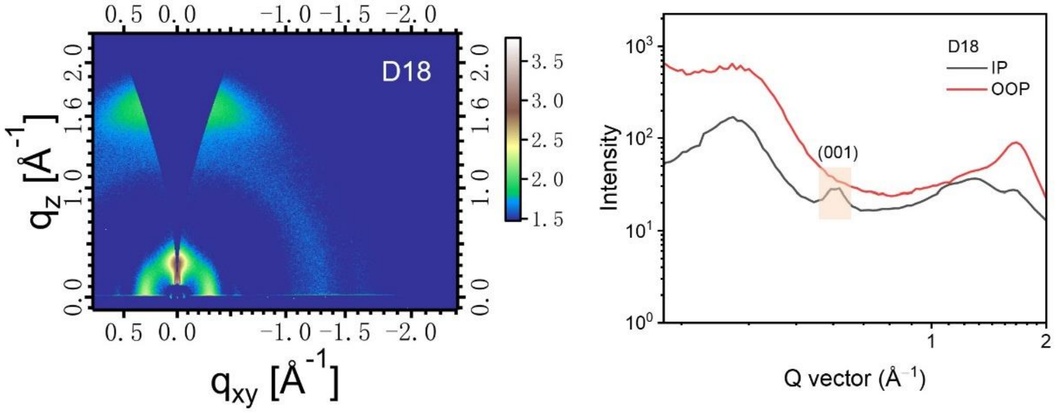

Supplementary Figure 25. GIWAXS 2D patterns and the corresponding line-cuts of GIWAXS patterns for D18 neat film.

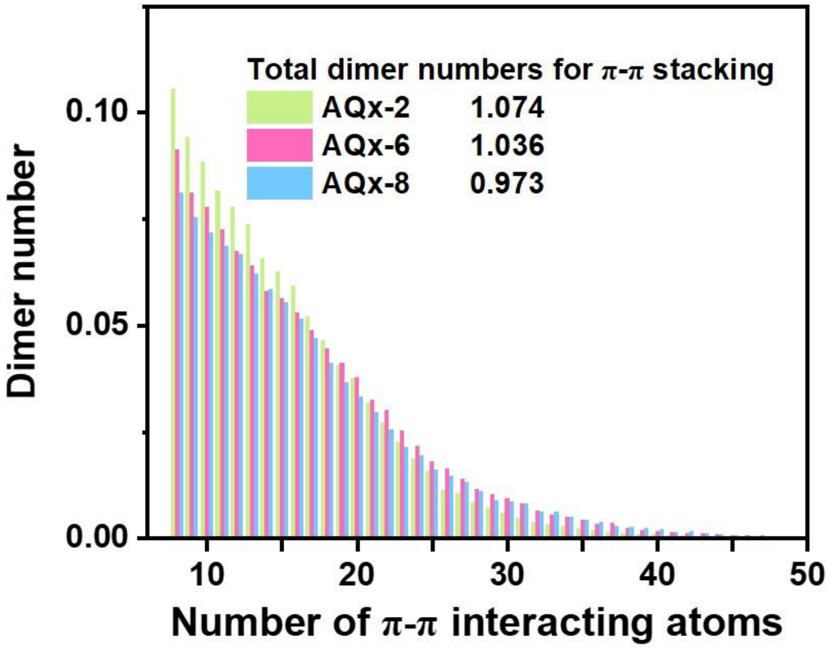

Supplementary Figure 26. The average number of dimers per molecule as a function of the number of backbones interacting atoms for the MD simulated AQx-2, AQx-6 and AQx-8 thin films. The dimers with ≥ 8 interacting atoms can be regarded as π-π stacking.

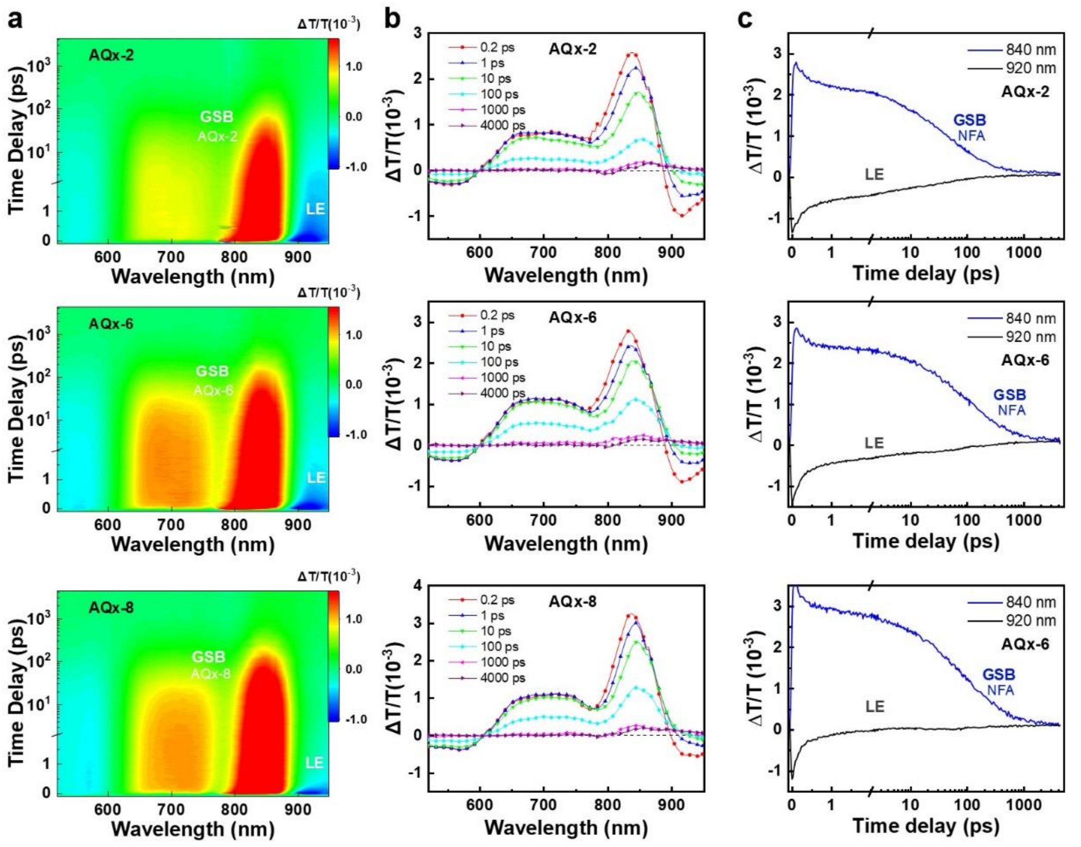

Supplementary Figure 27. a-c, TA profiles (a), TA spectra at different time delay (b) and TA traces of ground-states bleaching (GSB) signals probed at 840 nm and ESA signals of local excited (LE) state probed at 920 nm (c) for AQx-2, AQx-6 and:AQx-8 neat films with pump at 800 nm.

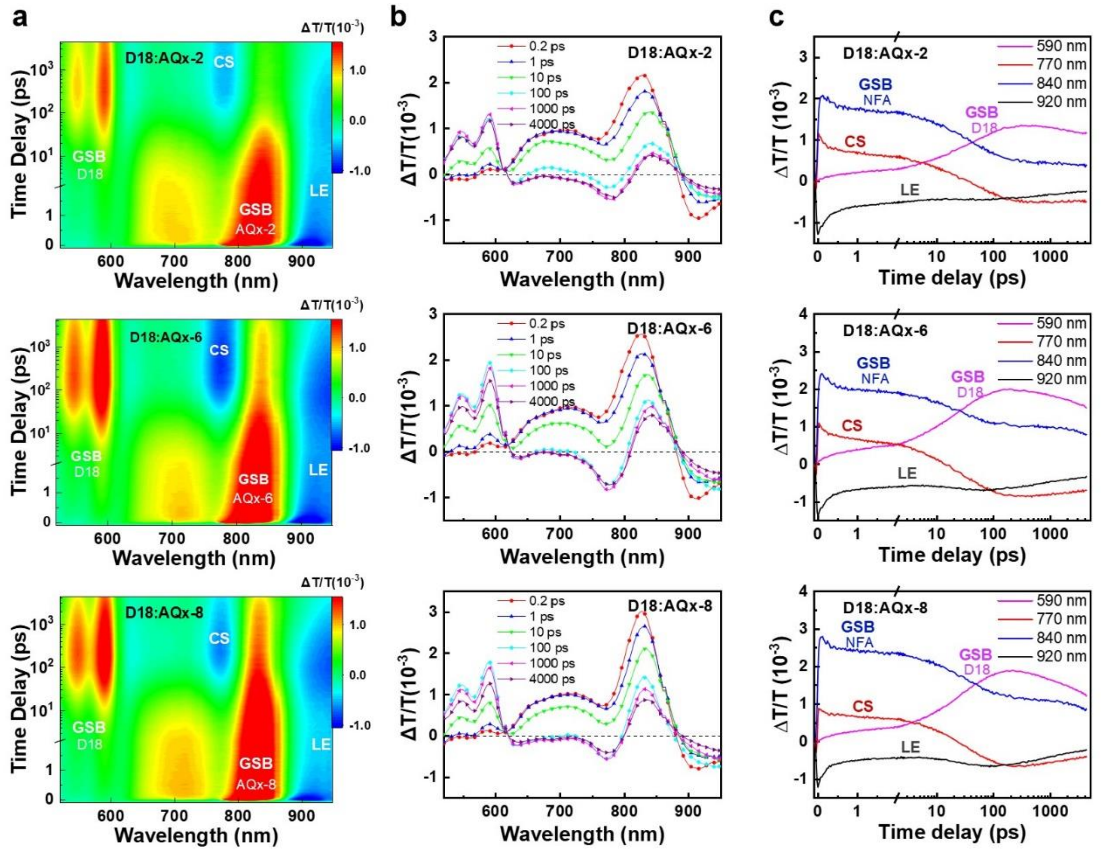

Supplementary Figure 28. a-c, TA profiles (a), TA spectra at different time delay (b) and TA traces of GSB signal for D18: 590nm, the CS signal for D18:AQx-6 blend: 770 nm, the GSB (840 nm) and LE signals (920 nm) of AQx-type acceptor (c) for AQx-2, AQx-6 and:AQx-8 neat films with pump at 800 nm.

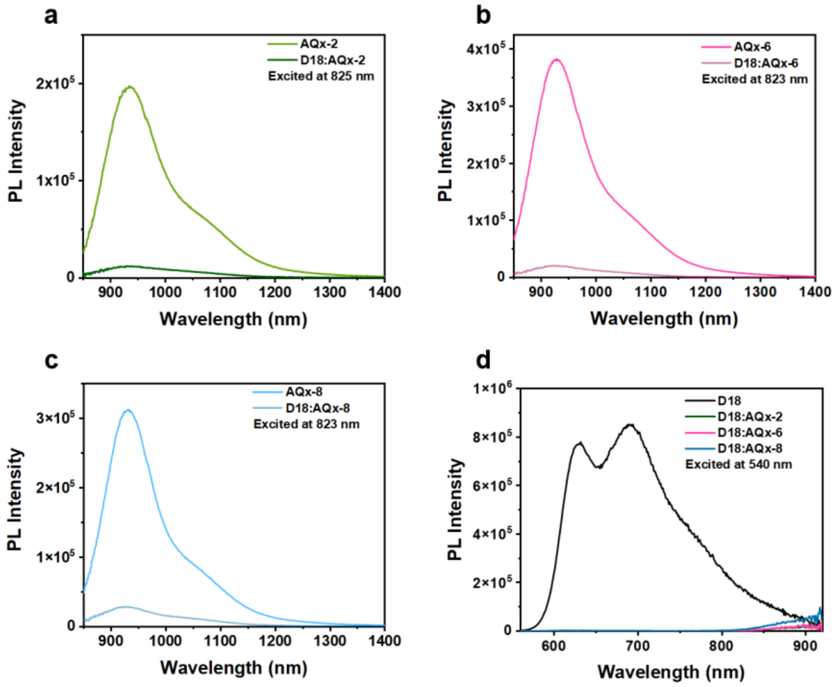

Supplementary Figure 29. Photoluminescence spectra for a-c) the AQx-type neat films and the corresponding blend films and d) the D18 film and the relevant blend films.

Compound 2.

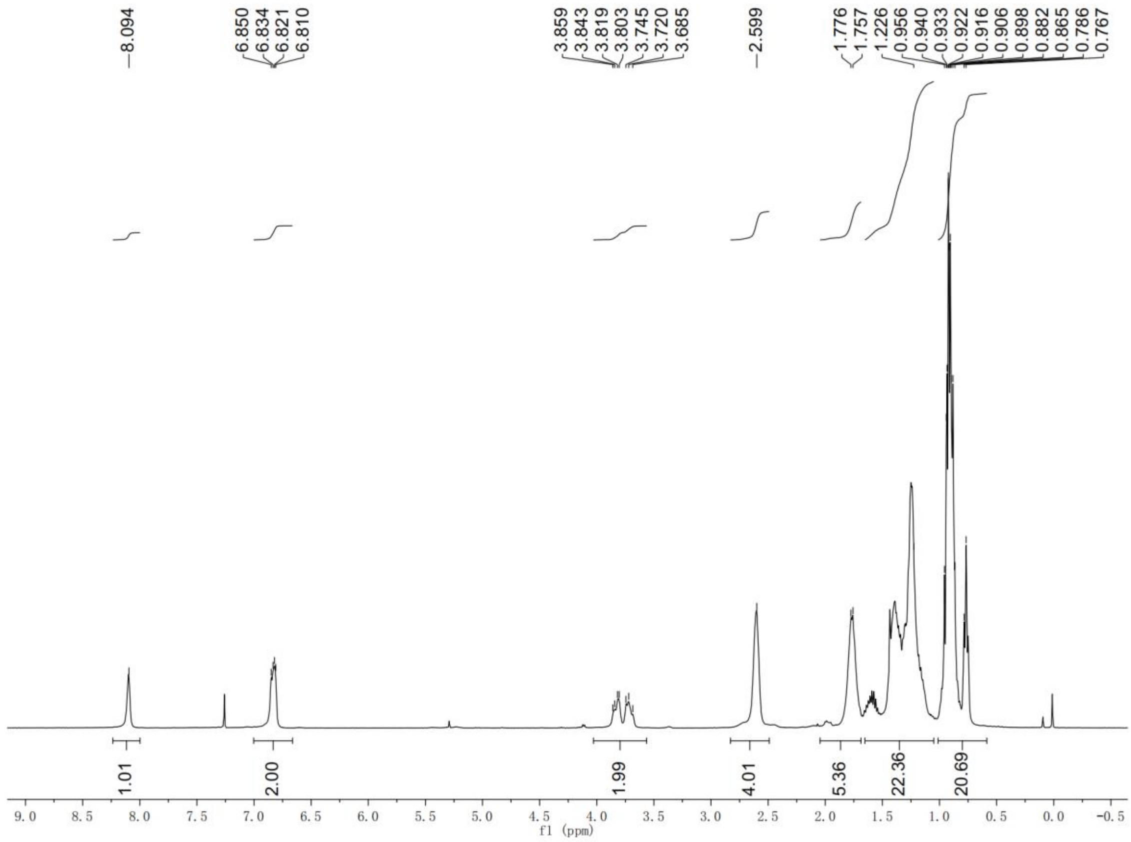

Supplementary Figure 30. The ^{1}H NMR spectrum for compound 2.

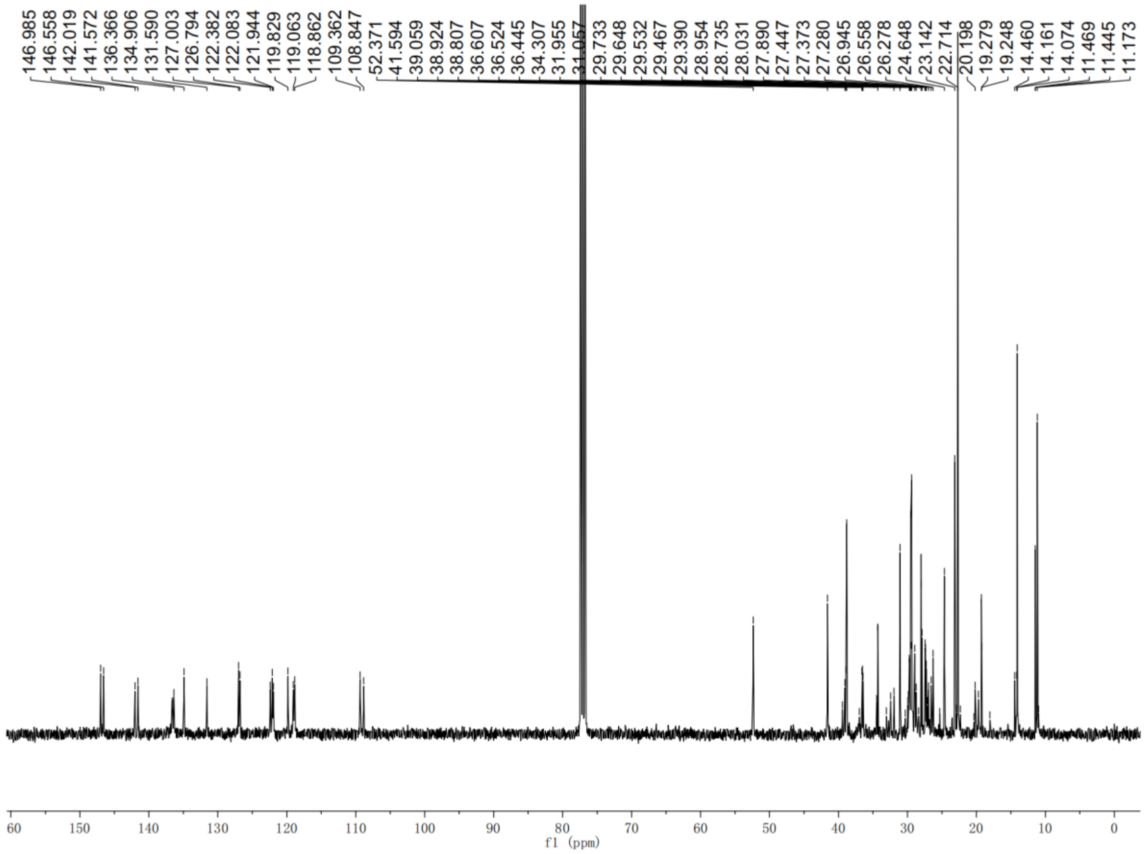

Supplementary Figure 31. The ^{13}C NMR spectrum for compound 2.

Compound 3.

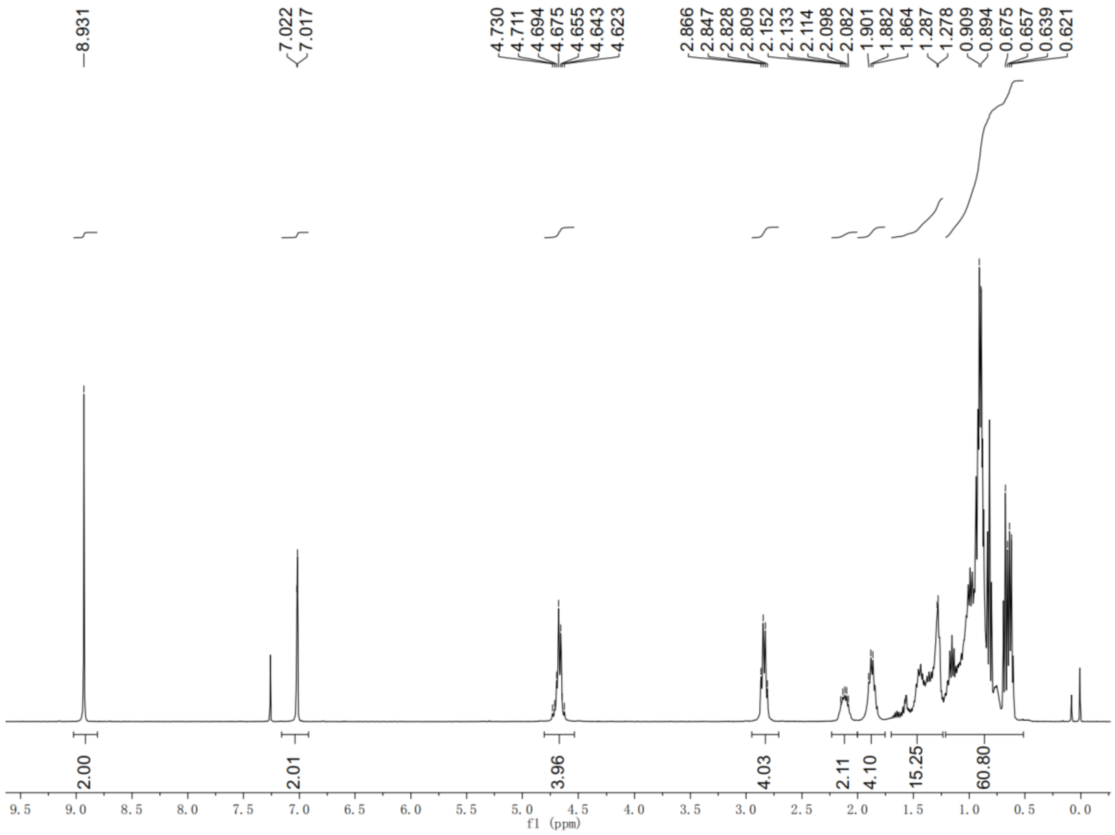

Supplementary Figure 32. The ^{1}H NMR spectrum for compound 3.

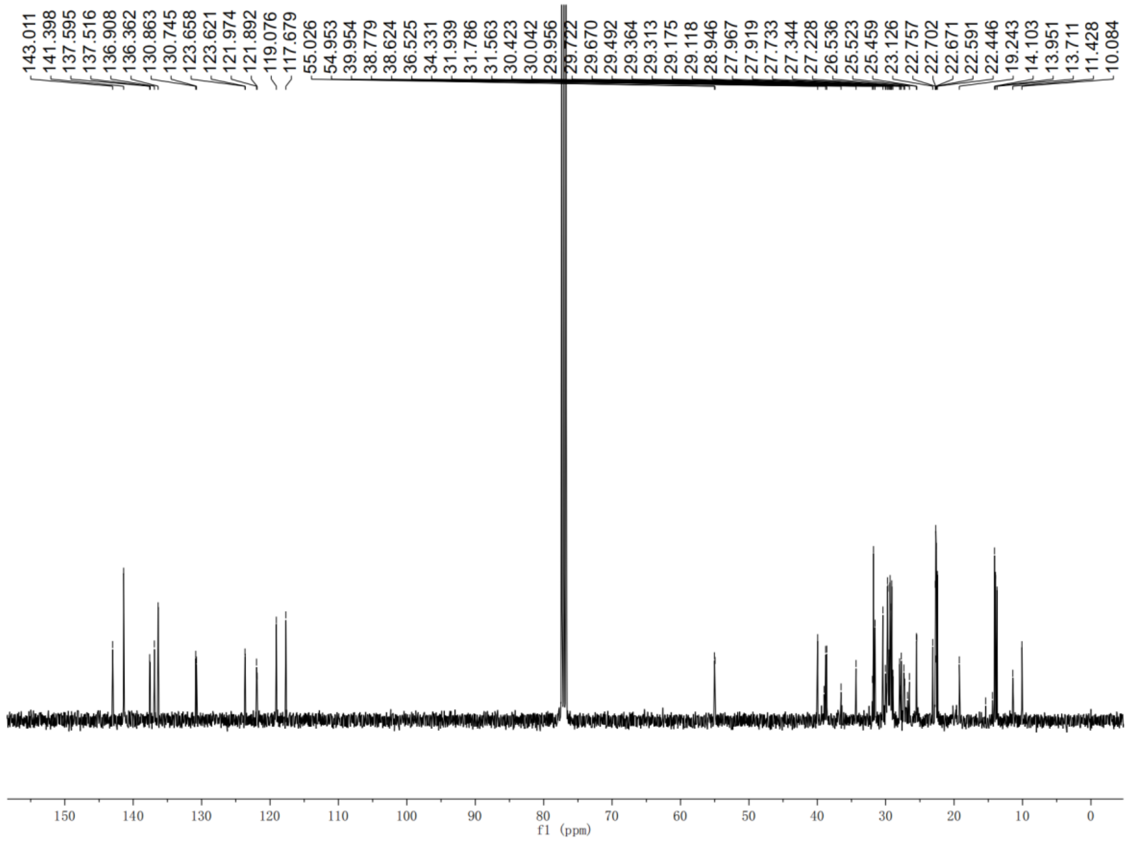

Supplementary Figure 33. The ^{13}C NMR spectrum for compound 3.

Compound 4.

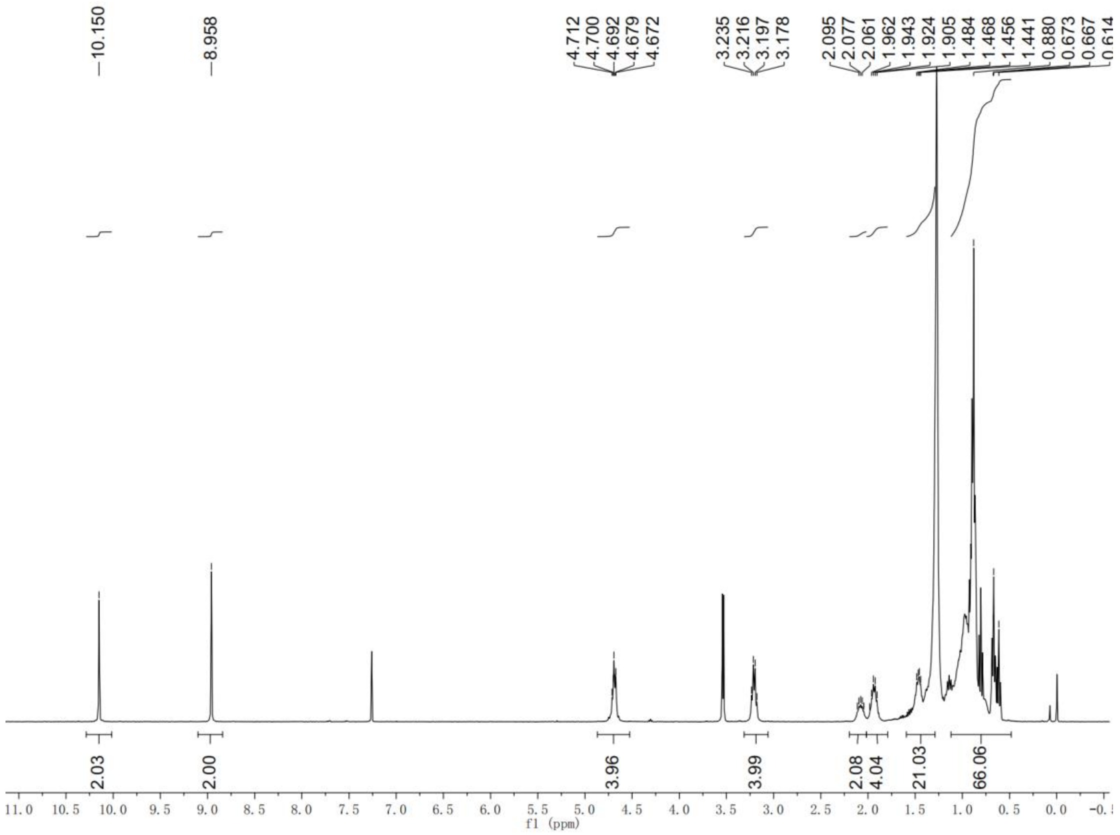

Supplementary Figure 34. The ^{1}H NMR spectrum for compound 4.

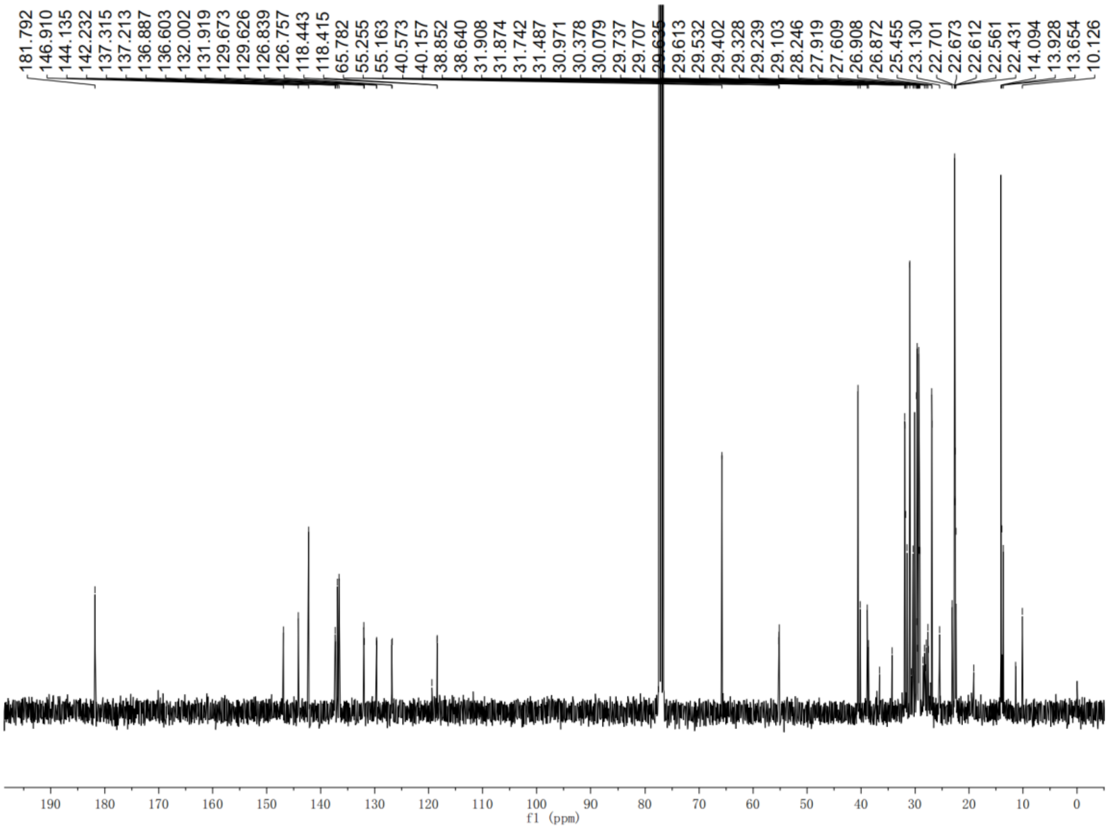

Supplementary Figure 35. The ^{13}C NMR spectrum for compound 4.

Compound AQX-6.

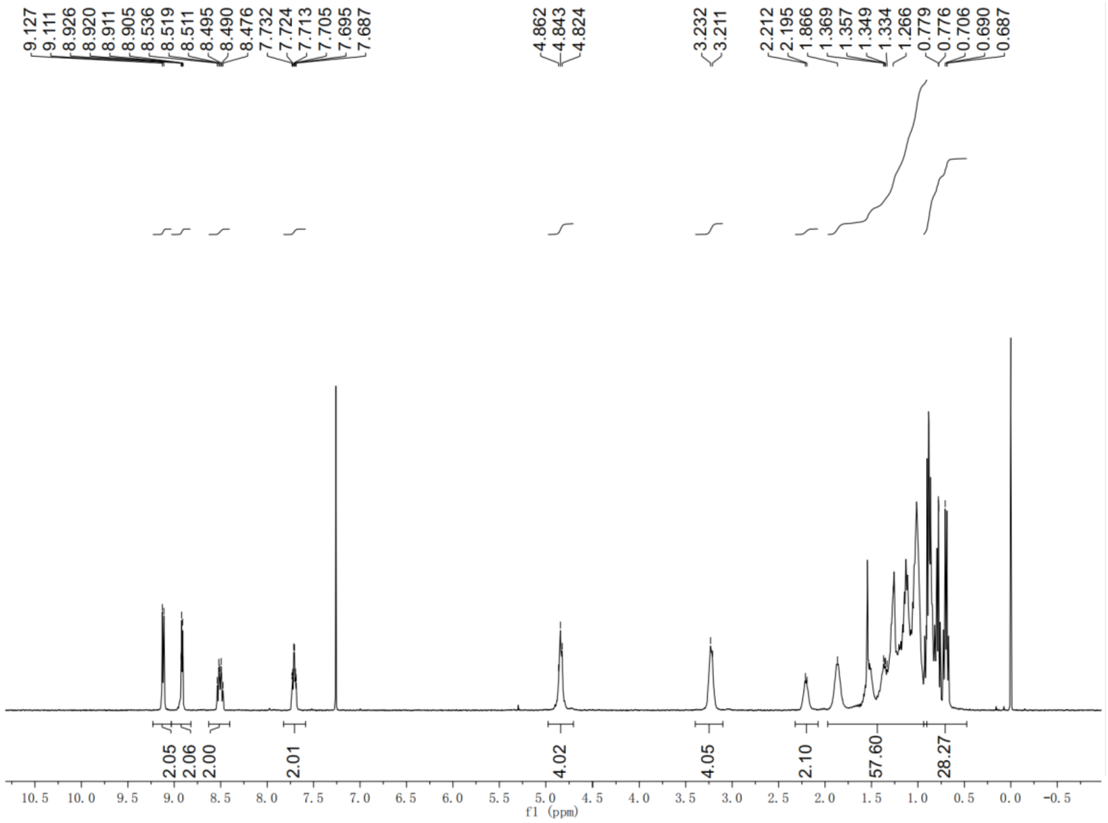

Supplementary Figure 36. The ^{1}H NMR spectrum for AQx-6.

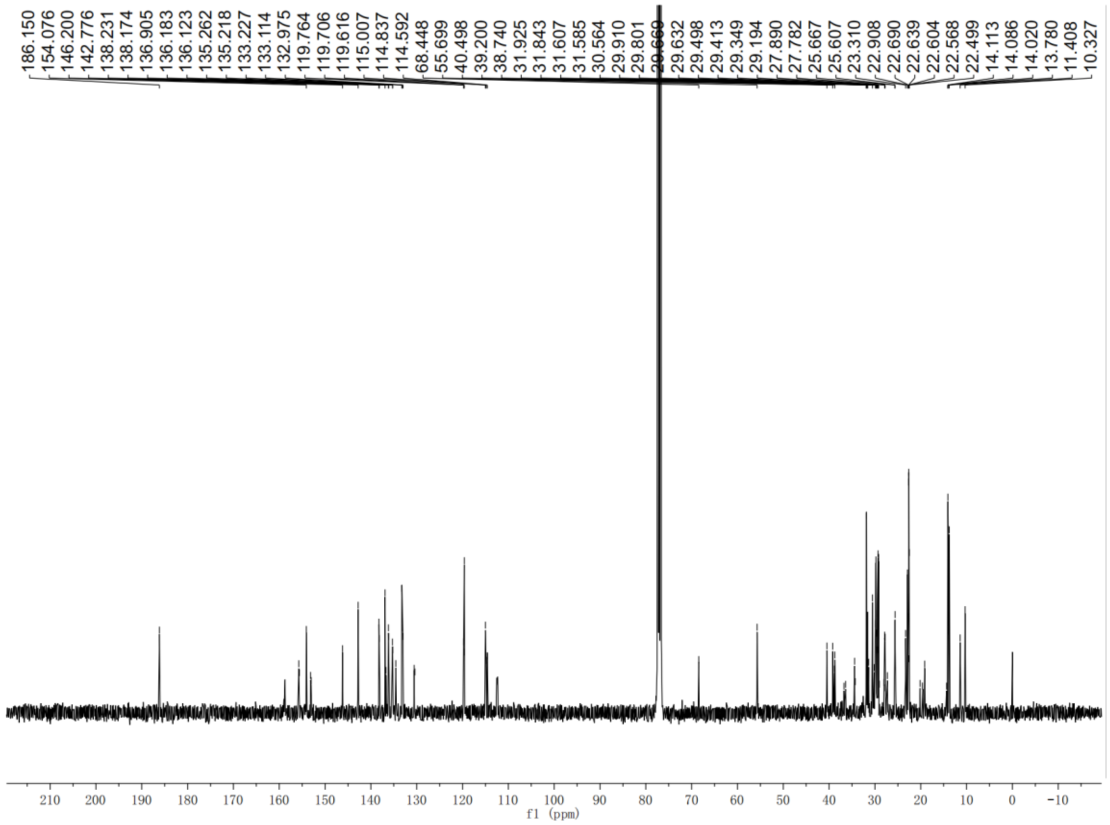

Supplementary Figure 37. The ^{13}C NMR spectrum for AQx-6.

Compound AQX-8.

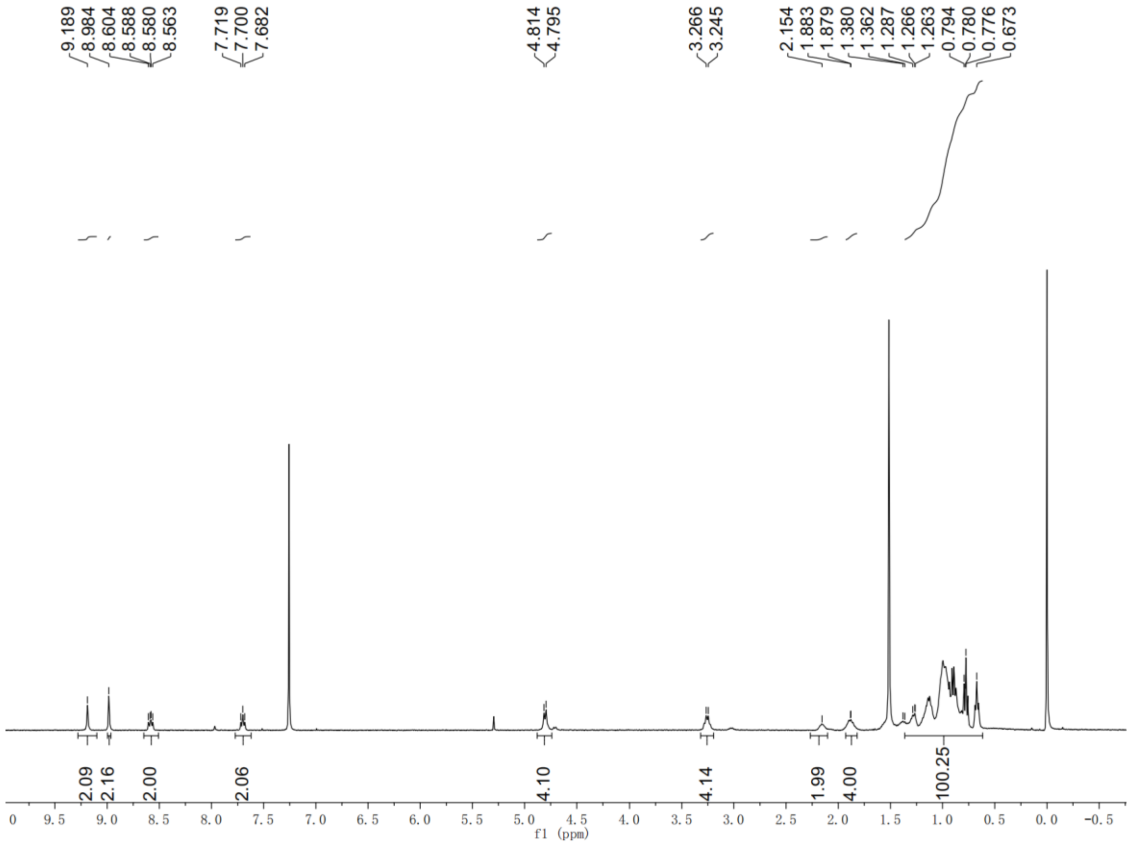

Supplementary Figure 38. The ^{1}H NMR spectrum for AQx-8.

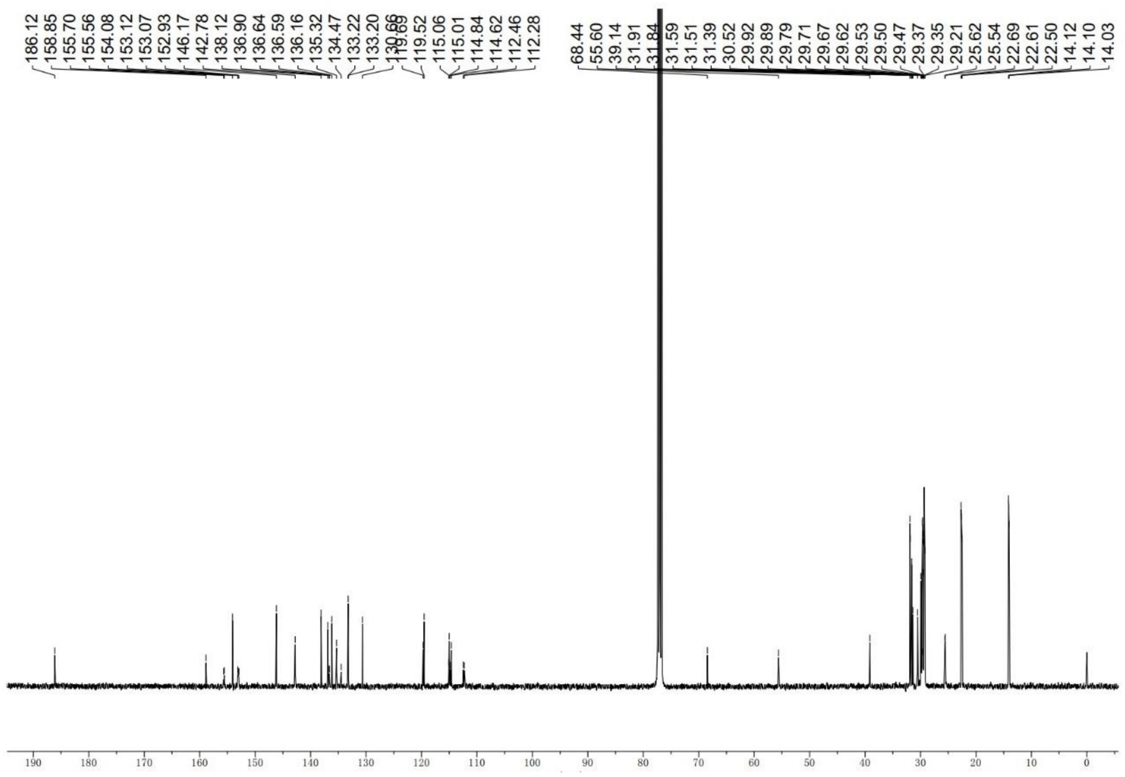

Supplementary Figure 39. The ^{13}C NMR spectrum for AQx-8.

3. Supplementary Tables

Supplementary Table 1. The PLQY, carrier lifetime (τ), radiative and nonradiative decay rate (k_r and k_{nr}) and molecular overlap for AQx-2, AQx-6, AQx-8.

Film	PLQY (%)	τ (ps)	k_r (s^{-1})	k_{nr} (s^{-1})	Molecular overlap
AQx-2	0.39	60.99	6.40×10^7	1.63×10^{10}	0.874
AQx-6	0.64	104.07	6.44×10^7	9.55×10^9	0.863
AQx-8	1.54	103.45	1.49×10^8	9.52×10^9	0.813

Supplementary Table 2. Photovoltaic parameters of OSCs based on D18:AQx-6 treated with different thermal annealing.

Treatment	V_{oc} [V]	J_{sc} [mA/cm^2]	FF [%]	PCE [%]
As cast	0.896	25.1	74.9	16.8
60°C	0.891	25.8	77.1	17.7
80°C	0.891	26.8	77.8	18.6
100°C	0.890	26.1	75.2	17.4
120°C	0.884	26.5	74.7	17.6

Supplementary Table 3. Photovoltaic parameters of OSCs based on D18:AQx-6 with different D/A ratio at 80°C thermal annealing.

D:A	V_{oc} [V]	J_{sc} [mA/cm^2]	FF [%]	PCE [%]
1:1	0.891	25.4	76.2	17.3
1:1.2	0.892	26.8	77.8	18.6
1:1.4	0.891	25.3	73.9	16.7

Supplementary Table 4. Photovoltaic parameters of OSCs based on D18:AQx-2 treated with different thermal annealing.

Treatment	V_{oc} [V]	J_{sc} [mA/cm^2]	FF [%]	PCE [%]
As cast	0.873	24.5	75.3	16.1
60°C	0.871	25.4	75.3	16.7
80°C	0.868	26.1	76.0	17.2
100°C	0.865	25.9	75.6	16.9
120°C	0.860	25.8	75.5	16.8

Supplementary Table 5. Photovoltaic parameters of OSCs based on D18:AQx-2 with different D/A ratio at 80°C thermal annealing.

D:A	V_{oc} [V]	J_{sc} [mA/cm^2]	FF [%]	PCE [%]
1:1	0.870	25.3	76.3	16.8
1:1.2	0.868	26.1	76.0	17.2
1:1.4	0.868	25.9	75.3	16.9

Supplementary Table 6. Photovoltaic parameters of OSCs based on D18:AQx-8 treated with different thermal annealing.

Treatment	V_{oc} [V]	J_{sc} [mA/cm^2]	FF [%]	PCE [%]
As cast	0.914	24.3	75.0	16.7
60°C	0.914	24.9	74.4	17.0
80°C	0.913	24.7	75.7	17.1
100°C	0.909	24.8	74.8	16.9
120°C	0.902	24.9	74.0	16.6

Supplementary Table 7. Photovoltaic parameters of OSCs based on D18:AQx-8 with different D/A ratio at 80°C thermal annealing.

D:A	V_{oc} [V]	J_{sc} [mA/cm^2]	FF [%]	PCE [%]
1:1	0.914	24.7	74.4	16.8
1:1.2	0.913	24.7	75.7	17.1
1:1.4	0.909	24.9	74.5	16.8

Supplementary Table 8. The E_{loss} of D18:AQx-2, D18:AQx-6 and D18:AQx-8 based OSCs.

Active layer	E_g [eV]	ΔE_{loss} [eV]	ΔE_1 [eV]	EQE_{EL}	ΔE_{nr} [eV]	ΔE_r [eV]
D18:AQx-2	1.397	0.529	0.265	3.29×10^{-4}	0.206	0.059
D18:AQx-6	1.410	0.518	0.266	4.05×10^{-4}	0.198	0.055
D18:AQx-8	1.406	0.493	0.266	8.65×10^{-4}	0.178	0.049

Supplementary Table 9. Surface energy of the D18, AQx-2, AQx-6 and AQx-8 pristine films.

Materials	θ_{DIM} [deg]	θ_{water} [deg]	γ [mN m^{-1}]	γ^p [mN m^{-1}]	γ^d [mN m^{-1}]	χ_{D-A}
D18	50.07	101.68	37.81	0.36	37.56	
AQx-2	35.53	86.56	42.00	0.323	41.681	0.11K
AQx-6	40.86	94.06	40.35	0.004	40.34	0.04K
AQx-8	41.37	96.24	40.61	0.082	40.53	0.05K

Supplementary Table 10. The peak position and CCL of different peaks o of pristine AQx-2, AQx-6 and AQx-8 films.

Film	Direction	Q ($Å^{-1}$)	Stacking Distance (Å)	FWHM ($Å^{-1}$)	CCL (Å)
AQx-2	IP	0.274	22.920	0.11	51.38
		0.403	15.583	0.16	35.55
		1.240	5.065	0.33	17.13
	OOP	1.520	4.132	1.21	4.67
		1.743	3.605	0.28	20.48
AQx-6	IP	0.368	17.065	0.19	30.39
		1.230	5.105	0.34	16.62
	OOP	1.436	4.373	1.31	4.3`
		1.737	3.615	0.29	19.76
AQx-8	IP	0.313	20.064	0.09	63.51
		1.280	4.906	0.47	12.03
	OOP	1.521	4.129	0.785	7.20
		1.704	3.685	0.272	20.779

Supplementary Table 11. The peak position and CCL of different peaks o of D18:AQx-2, D18:AQx-6 and D18:AQx-8 blend films.

Film	Direction	Q ($Å^{-1}$)	Stacking Distance (Å)	FWHM ($Å^{-1}$)	CCL (Å)
D18:AQx-2	IP	0.303	20.726	0.08	69.78
		0.518	12.126	0.22	26.29
		1.250	5.024	0.40	14.13
	OOP	1.472	4.266	0.94	6.03
		1.715	3.662	0.23	25.01
D18:AQx-6	IP	0.310	20.258	0.08	75.36
		0.543	11.565	0.14	41.56
		1.250	5.024	0.40	14.13
	OOP	1.450	4.331	1.06	5.35
		1.713	3.666	0.28	20.11
D18:AQx-8	IP	0.341	18.416	0.16	35.33
		1.260	4.984	0.40	14.13
	OOP	1.455	4.316	0.82	6.893
		1.714	3.664	0.32	17.66

Supplementary Table 12. Statistical sheet of PCE- ΔE_{nr} data of bulk- heterojunction OSCs reported in recent year.

Active layer	ΔE_{nr} (eV)	PCE (%)	Reference
PBDB-TF:AQx-2	0.22	16.64	*Adv. Mater.* **2020**, 32, 1906324.
PM6:Y6	0.217	16.00	*Adv. Mater.* **2019**, 31, 1905645.
S1:Y6	0.23	16.40	*Energy Environ. Sci.* **2019**, 12, 3328.
PBDB-TF:BTP-4Cl	0.206	16.50	*Nat. Commun.* **2019**, 10, 2515.
ZR1:Y6	0.24	14.34	*Nat. Commun.* **2019**, 10, 5393.
PM6:Y11(130)	0.201	16.50	*Nat. Photonics.* **2020**, 14, 300.
PM6:Y11(150)	0.202	16.54	
PBQx-TF:eC9-2Cl	0.229	17.7	*Adv. Mater.* **2021**, 33, 2102420.
PM6:BTP-S2	0.22	16.37	*Adv. Mater.* **2020**, 32, 2001160.
PBDTT1Cl:Y18-1F	0.19	17.1	*Adv. Mater.* **2021**, 2105483.
PTQ10:Y6	0.23	16.21	*Adv. Mater.* **2019**, 31, 1905480.
PBDB-TF:BTP-eC9	0.227	17.8	*Adv. Mater.* **2020**, 1908205
PM6:L8-BO	0.24	18.32	*Nat. Energy.* **2021**, 6, 605.
PM6:BTP-S2	0.2067	17.79	*Energy Environ. Sci.* **2022**.
PBDB-T-2F:BTP-4F-P3EH	0.19	18.22	*Adv. Energy Mater.* **2021**, 11, 2102596.
PM6:BTP:C9-N4F	0.2	17	*Sol. RRL.* **2021**,5, 2100008.
PM6:BTP-S2	0.22	16.37	*Adv. Mater.* **2020**, 32, 2001160.
PM6:AC9	0.238	18.34	*Adv. Funct. Mater.* **2022**, 2112511.
PM6:BTP-eC9	0.251	18.11	
PM6:Qx-2	0.190	18.22	*Nat. Commun.* **2022**, 13, 3256.

PM6:CH6	0.23	18.33	*Angew. Chem.Int. Ed.* 2022,61, 202209580.
PM6:2BTP-2F-T	0.199	18.18	*Adv. Sci.***2022**, 2202513.
PM6:AC9	0.243	18.43	*Adv. Funct. Mater.* **2022**, 2112511.
PM6:mPh4F-TS	0.213	18.05	*Angew. Chem.Int. Ed.***2022**,61, e2022.
PM6:BOEH-4Cl	0.244	17.4	*J. Mater. Chem. A* **2022**, 10, 21061–21071.
PM6:BO-4Cl	0.229	17.43	*Nat. Commun.* **2022**, 13, 2598.
