# Supplementary material for: Suppressing electron-phonon coupling in organic photovoltaics for high-efficiency power conversion
Source: Nat Commun. 2023 Aug 21;14:5079. doi: 10.1038/s41467-023-40806-9 (PMC10442373; doi:10.1038/s41467-023-40806-9)
Supplement: Supplementary file 3 — Reporting Summary [file 41467_2023_40806_MOESM3_ESM.pdf]

nature research

Corresponding author(s): Shengjie Xu, Yuanping Yi, Xiaozhang Zhu

Solar Cells Reporting Summary

Nature Research wishes to improve the reproducibility of the work that we publish. This form is intended for publication with all accepted papers reporting the characterization of photovoltaic devices and provides structure for consistency and transparency in reporting. Some list items might not apply to an individual manuscript, but all fields must be completed for clarity.

For further information on Nature Research policies, including our data availability policy, see Authors & Referees.

Experimental design

Please check: are the following details reported in the manuscript?

1. Dimensions

Item	Answer	Details
Area of the tested solar cells	☒ Yes ☐ No	0.04 cm^2 in our lab and 0.03159 cm^2 in National Institute of Metrology (NIM), Chain.
Method used to determine the device area	☒ Yes ☐ No	Area of the tested solar cells in our lab is defined by optical microscope and area of the tested solar cells in NIM is defined by mask.

2. Current-voltage characterization

Item	Answer	Details
Current density-voltage (J-V) plots in both forward and backward direction	☐ Yes ☒ No	We only scan the device in forward direction. Generally there in no difference between these two direction for organic solar cell.
Voltage scan conditions<br>*For instance: scan direction, speed, dwell times*	☒ Yes ☐ No	Forward direction, the scan voltage from -1.5 to 1.5 V with a voltage step of 10 mV and delay time of 1ms.
Test environment<br>*For instance: characterization temperature, in air or in glove box*	☒ Yes ☐ No	Our devices were characterized at room temperature (ca. 25 Celsius degree) in glove box.
Protocol for preconditioning of the device before its characterization	☐ Yes ☒ No	No preconditioning protocol.
Stability of the J-V characteristic<br>*Verified with time evolution of the maximum power point or with the photocurrent at maximum power point; see ref. 7 for details.*	☐ Yes ☒ No	Organic solar cells show no decay or instability during the test of J-V characteristics.

3. Hysteresis or any other unusual behaviour

Item	Answer	Details
Description of the unusual behaviour observed during the characterization	☐ Yes ☒ No	No hysteresis or other unusual behaviour was observed during the characterization of the solar cells. In general, organic solar cells do not have hystereisis problems.
Related experimental data	☐ Yes ☒ No	No hysteresis or other unusual behaviour was observed during the characterization of the solar cells.

4. Efficiency

Item	Answer	Details
External quantum efficiency (EQE) or incident photons to current efficiency (IPCE)	☒ Yes ☐ No	The EQE curves are shown in Figure 3b, which is obtained by the equipment of Enlitech (Taiwan) with a standard Si cell in air condition.
A comparison between the integrated response under the standard reference spectrum and the response measure under the simulator	☒ Yes ☐ No	The difference between the integrated current density from EQE and the short-circuit current density from J-V is within 5%.
For tandem solar cells, the bias illumination and bias voltage used for each subcell	☐ Yes ☒ No	Our devices were only fabricated for single-junction solar cells.

5. Calibration

Item	Answer	Details
Light source and reference cell or sensor used for the characterization	☒ Yes ☐ No	The illumination of AM 1.5G (100mW cm-2) was achieved by a XES-70S1 solar simulator (SAN-EI Electric Co., Ltd., AAA grade, 70 mm × 70 mm photo-beam size).
Confirmation that the reference cell was calibrated and certified	☒ Yes ☐ No	The reference cell was calibrated an certified in NIM.

Calculation of spectral mismatch between the reference cell and the devices under test

☐ Yes
☒ No

The spectral mismatch factor was determined at National Institute of Metrology, China (NIM). We do not have the detailed information for the method.

6. Mask/aperture

Size of the mask/aperture used during testing

☐ Yes
☒ No

We did not use a mask during testing in our lab.

Variation of the measured short-circuit current density with the mask/aperture area

☐ Yes
☒ No

We did not use a mask during testing in our lab.

7. Performance certification

Identity of the independent certification laboratory that confirmed the photovoltaic performance

☒ Yes
☐ No

The certified PCE was obtained from the National Institute of Metrology (NIM), China.

A copy of any certificate(s)
Provide in Supplementary Information

☒ Yes
☐ No

Supplementary Figure 14

8. Statistics

Number of solar cells tested

☒ Yes
☐ No

The average PCE of the devices is obtained from ten independent devices.

Statistical analysis of the device performance

☒ Yes
☐ No

We have given statistical data of device performance in Table 1.

9. Long-term stability analysis

Type of analysis, bias conditions and environmental conditions
For instance: illumination type, temperature, atmosphere humidity, encapsulation method, preconditioning temperature

☐ Yes
☒ No

We did not perform the long-term stability test.
